# Heteroleptic Copper(I)-Based Complexes Incorporating BINAP and π-Extended Diimines: Synthesis, Catalysis and Biological Applications

**DOI:** 10.3390/molecules27123745

**Published:** 2022-06-10

**Authors:** Corentin Cruché, Sayak Gupta, Jeremy Kodanko, Shawn K. Collins

**Affiliations:** 1 Département de Chimie, Centre for Green Chemistry and Catalysis, Université de Montréal, 1375 Avenue Thérèse-Lavoie-Roux, Montréal, QC H2V 0B3, Canada; corentin.cruche@umontreal.ca; 2 Department of Chemistry, Wayne State University, 5101 Cass Ave., Detroit, MI 48202, USA; gu8510@wayne.edu

**Keywords:** copper, photochemistry, medicinal chemistry

## Abstract

A series of copper-based photocatalysts of the type Cu(**NN**)(**BINAP**)BF_4_ were synthesized bearing π-extended diimine ligands. Their behavior in several photocatalytic processes were evaluated and revealed acceptable levels of activity in an SET process, but negligible activity in PCET or ET processes. Suitable activity in ET processes could be restored through modification of the ligand. The BINAP-derived complexes were then evaluated for activity against triple-negative breast cancer cell lines. Controls indicated that copper complexes, and not their ligands, were responsible for activity. Encouraging activity was displayed by a homoleptic complex Cu(**dppz**)_2_BF_4_.

## 1. Introduction

Copper-based complexes have demonstrated their potential across photocatalysis [1]. As an alternative to precious metal complexes [2,3,4], discreet copper-based complexes can be exploited as photocatalysts under UV [5,6,7] and visible-light irradiation [8,9,10,11,12]. In turn, a number of copper complexes can be formed in situ and used in metallaphotoredox processes, which are particularly advantageous for asymmetric photocatalysis [13]. In addition to synthetic applications, copper-based complexes have found interest in solar energy sciences [14], photocatalytic water splitting [15] and organic light-emitting diodes [16].

**Figure 1:**
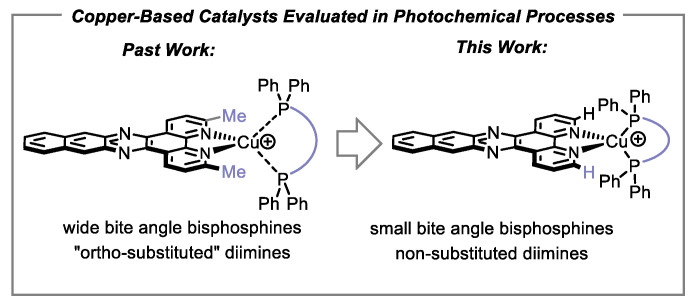
Small-bite-angle bisphosphine for heteroleptic copper-based complexes.

McMillin and co-workers first reported that heteroleptic copper-based complexes (Cu(**NN**)(**PP**)X) bearing the wide-bite-angle bisphosphine **DPEPhos** possessed unusually long excited-state lifetimes [17]. Following our initial discovery that such complexes exhibited significant potential for synthetic photocatalysis [18], we reported a structure/activity study library of 50 copper complexes that were evaluated in single-electron transfer (SET) [19,20], energy transfer (ET) [21] and proton-coupled electron transfer (PCET) reactions [22]. Although most photocatalysis using heteroleptic complexes continue to employ wide-bite-angle bisphosphines [23,24], the aforementioned library study revealed that the small-bite-angle bisphosphine **BINAP** could form copper complexes that afforded high yields in all three of the mechanistic processes evaluated. Our bank of available diimines and bisphosphines has since expanded to allow for development of improved complexes for energy transfer processes [25]. Among the diimine structures evaluated were those that possessed extended π-surfaces, which unfortunately did not afford heteroleptic complexes with remarkable activities in photocatalysis [26]. However, previous studies were limited to wide-bite-angle bisphosphines, as the preparation of the corresponding complexes with BINAP was problematic (Figure 1). Herein, we describe the synthesis of the “missing” copper-based complexes of the type Cu(**NN**)(**BINAP**)BF_4_, their evaluation in photochemical processes and preliminary biological testing against triple-negative breast cancer cell lines.

## 2. Results and Discussion

**Figure 2:**
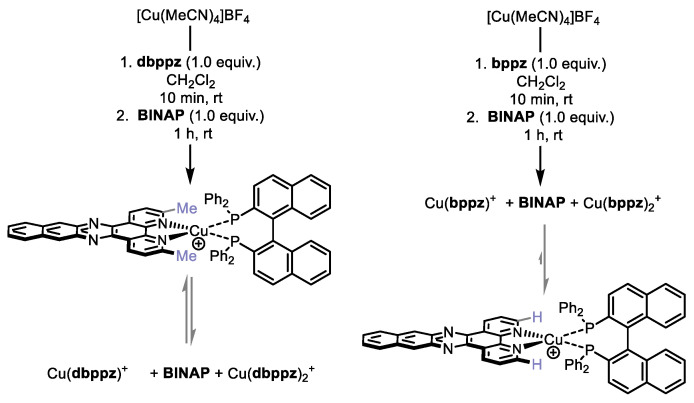
Synthesis of heteroleptic copper-based complexes using BINAP.

Heteroleptic complexes are typically formed by sequential addition of the diimine and bisphosphine ligands to a copper salt in a solvent, followed by precipitation. When the synthesis of heteroleptic Cu(I)-based photocatalysts using **BINAP** and the ligands **ddpq**, **ddppz**, or **dbdppz**^−^ [27] was attempted, the resulting solids were mixtures of the corresponding hetero- and homoleptic complexes (^1^H-NMR and mass spectrometry, see supplementary materials) (Figure 2). Our hypothesis was that the small-bite-angle oriented the phenyl groups of the phosphine over the copper center, which is already encumbered by the methyl groups found on the diimine ligands. Attempts at conducting the synthesis in other solvents (PhME, THF, and mixtures thereof with CH_2_Cl_2_) did not result in a shift in the equilibrium between heteroleptic and homoleptic complexes. Experiments involving lower temperatures and/or slow addition of the bisphosphine were also non-productive. Repeated crystallization of crude reaction mixtures did improve the ratio of heteroleptic versus homoleptic complexes, but did not approach selectivities or yields that were synthetically useful.

Consequently, the **BINAP**-containing complexes were prepared with the analogous diimines **dpq**, **dppz**, and **bdppz** (Table 1). Gratifyingly, all three complexes were isolated in good yields (53–77%). When examining the photophysical data, the UV-vis absorption characteristics of the **BINAP**-containing photocatalysts did not change significantly with respect to the diimine. The absorption maxima are all within a narrow window (424–462 nm), although the emission maxima are more spread out (560–625 nm) with lower-wavelength emissions observed for the larger diimine ligands. Extinction coefficients and excited-state lifetimes are again all relatively similar across the series. The short excited-state lifetimes are to be expected, as the absence of both *ortho*-substitution on the diimines and the small bite angle of the bisphosphine will not stabilize the geometry of the excited state. Excited state reduction potentials all were in the range of ~1.0 eV which corresponds to what was observed with the complexes derived from ortho-substituted analogues having **ddpq**, **ddppz**, and **dbdppz** ligands.

**Figure 3:**
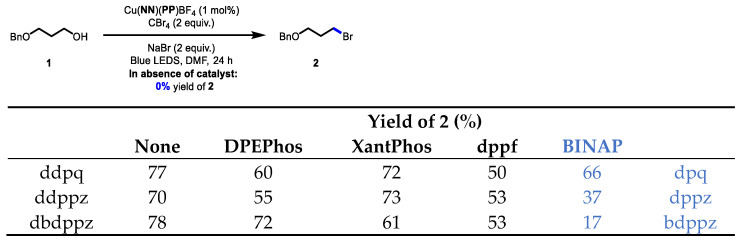
Comparison of the **BINAP**-containing copper complexes bearing π-extended ligands in a photochemical Appel-type process.

**Figure 4:**
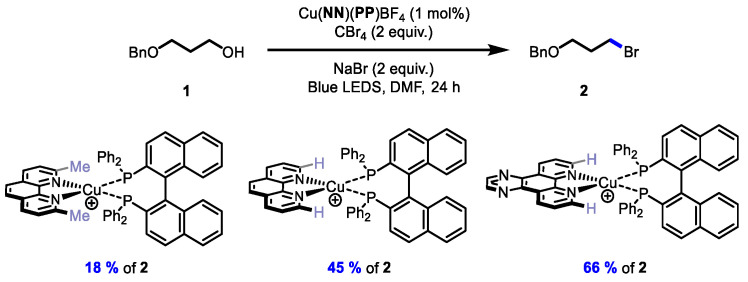
Effects of the diimine ligand of heteroleptic copper complexes in a photochemical Appel-type process.

With the new **BINAP**-containing copper-based complexes, their evaluation in photocatalysis was performed and compared to analogous catalysts. Three mechanistically distinct photocatalytic transformations were pursued. In a visible-light Appel-type reaction (Figure 3) [28,29], the new **BINAP**-containing copper-based catalyst Cu(**dpq**)(**BINAP**)BF_4_ provided similar yields to other complexes having large-bite-angle bisphosphines. Note that control reactions performed in the absence of light or in the absence of catalyst at 450 nm did not afford any significant conversion to the alkyl bromide **2**. However, as the π-surface of the ligands grew, the **BINAP**-containing complexes of **dppz** and **bdppz** were all inferior to analogous complexes having wide-bite-angle bisphosphines. Although the complexes of **dppz** and **bdppz** had larger excited-state reduction potentials, it is possible that the complexes with ligands with larger π-surfaces could be more unstable in solution. The stability of various copper complexes with diimines having large π-surfaces was previously shown to decrease with the size of the ligand in other photocatalytic processes [26]. However, it should be noted that amongst other **BINAP**-derived complexes, the Cu(**dpq**)(**BINAP**)BF_4_ (66% of **2**) was superior in the Appel-type reaction to other structurally similar complexes such as Cu(**dmp**)(**BINAP**)BF_4_ (18% of **2**) and Cu(**phen**)(**BINAP**)BF_4_ (45% of **2**), suggesting that the **dpq** offered some beneficial reactivity (Figure 4).

**Figure 5:**
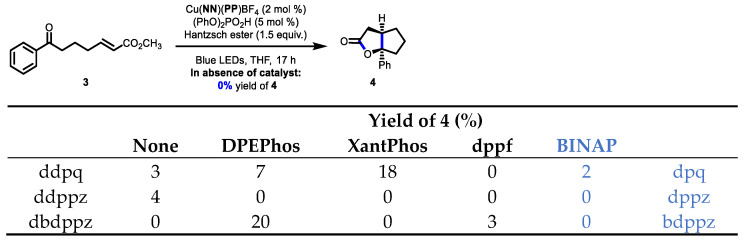
Comparison of the **BINAP**-containing copper complexes bearing π-extended ligands in a photochemical PCET process.

The **BINAP**-containing catalysts were then compared to the previous series being the ortho-substituted diimines in a reductive proton-coupled electron transfer (PCET) reaction. Our group has previously used the homolytic activation of ketones to benchmark complexes for their efficiency in a PCET process (Figure 5) [30] Previous evaluation with the ortho-substituted series revealed very poor reactivity and low yields (0–20% yield). Unfortunately, the screening with the new **BINAP**-containing complexes was equally disappointing. Recent work suggests that the process is in fact a reductive quenching of the Cu-based photocatalysts in the excited state [31,32] The electron-rich π-extended ligands would not be favorable in such a mechanism. Furthermore, given the results from the oxidative quenching in the Appel process, it is clear that the bisphosphine is not playing a significant role in altering the excited-state redox potentials of the resulting complexes.

**Figure 6:**
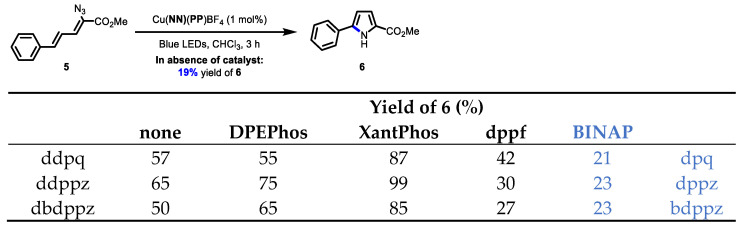
Comparison of the **BINAP**-containing copper complexes bearing π-extended ligands in an energy transfer process.

**Figure 7:**
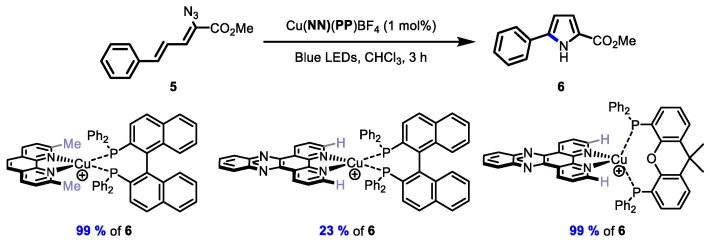
Ligand effects in heteroleptic copper complexes in an energy transfer process.

The last evaluation of the new **BINAP**-containing Cu-based photocatalysts was via energy transfer for the transformation of vinyl azides to the corresponding pyrrole (Figure 6) [33]. Given that the new complexes had neither wide-bite-angle phosphines or ortho-substituted diimines to stabilize the excited state, the yields of the pyrrole were expected to drop. Note that the excited-state lifetimes of the new **BINAP**-containing complexes were all approximately an order of magnitude less than analogous complexes (e.g., Cu(**bdppz**)(**XantPhos**)BF_4_ τ = 71 ns; Cu(**dppz**)(**BINAP**)BF_4_ τ = 1.8 ns). Indeed, the yields of the pyrrole **6** with the **BINAP**-containing complexes (21–23% of **6**) were barely above the observed background reaction in the absence of any catalyst at 450 nm (19% of **6**). A further comparison of Cu(**phen**)(**BINAP**)BF_4_ (38% of **6**) and Cu(**dpq**)(**BINAP**)BF_4_ (21% of **6**) showed that the **dpq** ligand had a deleterious effect on the energy transfer process. It should be noted that good yields of the pyrrole are possible when switching to any ligand known to extend the excited-state lifetimes (Figure 7). For example, using an ortho-substituted diimine ligand in a complex with **BINAP** affords quantitative yields of the product (Cu(**dmp**)(**BINAP**)BF_4_, 99% of **6**). In addition, using a wide-bite-angle bisphosphine also affords a quantitative yield of **6** (Cu(**dppz**)(**XantPhos**)BF_4_, 99% of **6**).

**Figure 8:**
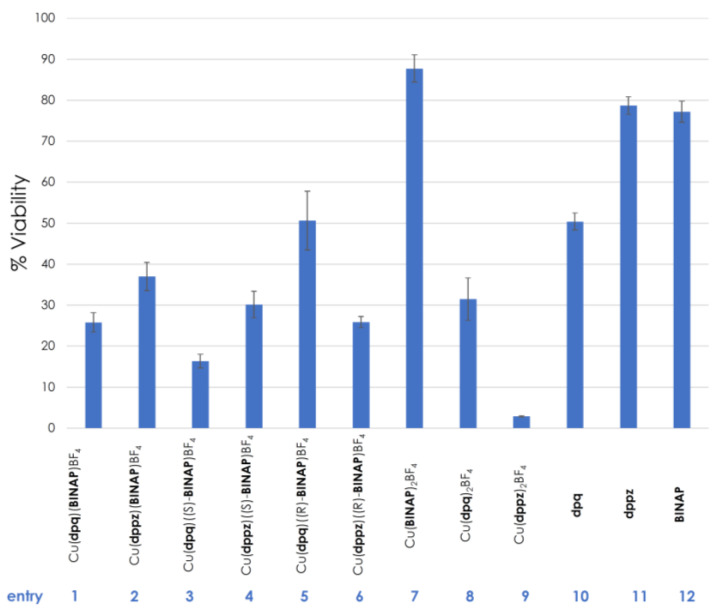
Viability of MDA-MB-231 cells at 5 µM. MDA-MB-231 cells were seeded at the density of 7000 cells per well in 96-well plates; plates were incubated overnight. The cells were then treated with growth media containing 5 µM of the copper complexes (entries **1–9**) or controls (entries **10**–**12**) and allowed to incubate at 37 °C for 72 h. The viabilities of cells were finally determined by MTT assay and converted to percentages. Data are an average of three different experiments.

Given the recent interest in copper-containing complexes for medicinal chemistry [34,35,36], it was decided to test the most soluble of the new **BINAP**-containing complexes against triple-negative breast cancer cell lines (MBA-MB-231) (Figure 8). The viability of the cell lines was evaluated with the Cu(**dpq**)(**BINAP**)BF_4_ and Cu(**dppz**)(**BINAP**)BF_4_ complexes (5 μM, entries 1 and 2, respectively) and both displayed approximately 25–35% viability. Controls performed from the **dpq**, **dppz**, and **BINAP** ligands (entries 10–12) demonstrated that biological activity was originating from the metal complexes themselves. While a homoleptic complex Cu(**BINAP**)_2_BF_4_ was poorly active, the homoleptic complexes derived from the diimines showed low cell viabilities, with Cu(**dppz**)_2_BF_4_ being the most active of all complexes tested. Finally, given that the **BINAP** used in the above photocatalysis and biological evaluations was racemic, we prepared and evaluated the enantiomer variants of the dpq- and dppz-containing complexes. Interestingly, for the dpq complexes, the (*S*)-**BINAP**-containing complex Cu(**dpq**)((*S*)-**BINAP**)BF_4_ was approximately twice as active as the analogous (*R*)-**BINAP** complex. The copper complexes of dppz bearing either (*S*)- or (*R*)-**BINAP** did not show any difference in activity. The complex [Cu(MeCN)_4_]BF_4_ (10 µM) had negligible effects on cell viability (>75%), indicating that the complexes, rather than free copper, were responsible for biological activity.

In summary, a series of copper-based photocatalysts of the type Cu(**NN**)(**BINAP**)BF_4_ were synthesized bearing π-extended diimine ligands. Their behavior in several photocatalytic processes was evaluated and revealed the following:

1. Copper-based complexes derived from **BINAP** with π-extended diimine ligands without ortho-substitution did not show significant different photophysical properties when compared to analogous complexes with the exception of the excited state lifetime, which decreased by approximately an order of magnitude.
2. The new **BINAP**-containing complexes were active in the visible-light Appel-type process, with the Cu(**dpq**)(**BINAP**)BF_4_ complex having slightly better activity than analogous complexes derived from **phen** of **dmp** ligands.
3. The new **BINAP**-derived complexes did not afford complexes active for a PCET process.
4. In an energy transfer process, high yields of the desired product could be obtained with either **BINAP** or the **dpq**, **dppz**, and **ddppz** diimines through judicious choice of the accompanying ligand. For example, Cu(**dmp**)(**BINAP**)BF_4_ and Cu(**dppz**)(**XantPhos**)BF_4_ afforded quantitative yields of the product.
5. In addition to the photocatalysis, the copper complexes were evaluated for the first time in a medicinal chemistry context against triple-negative breast cancer cell lines. Controls indicated that copper complexes, and not their ligands, were responsible for activity. Encouraging activity was displayed by a homoleptic complex Cu(**dppz**)_2_BF_4._

## Figures and Tables

**Table 1:** Synthesis and Properties of Cu(I)-Based Photocatalysts of the Type Cu(**NN**)(**BINAP**)BF_4_.

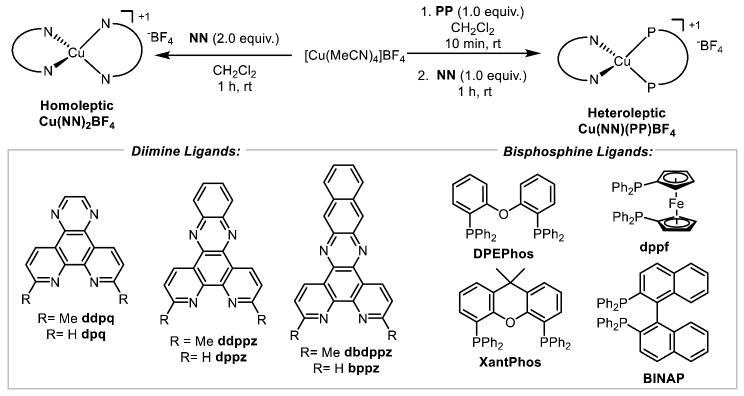									
Entry	NN	PP	Yield (%) *^a^*	_λ max_ (nm)	ε (L/mol·cm)	τ (ns)	_λ emm_ (nm)	E_T_ (eV)	E (* Cu^I^/Cu^II^)
1	ddpq		78	458	6760	3	680	1.82	−1.36
2	ddpq	DPEPhos	84	382	4485	5	565	2.19	−1.26
3	ddpq	XantPhos	85	386	3444	3	560	2.21	−1.72
4	ddpq	dppf	91	380	3346	73	530	2.34	−1.15
5	dpq	BINAP	75	424	5752	1.4	625	2.38	−1.02
6	ddppz		99	453	14428	4	762	1.63	−0.90
7	ddppz	DPEPhos	78	380	17508	44	664	1.87	−1.12
8	ddppz	XantPhos	91	380	12489	71	634	1.95	−0.82
9	ddppz	dppf	79	380	17508	61	510	2.43	−1.59
10	dppz	BINAP	77	433	6759	1.8	545	2.27	−1.26
11	dbdppz		82	412	25891	78	567	2.19	−1.34
12	dbdppz	DPEPhos	77	409	16663	69	489	2.53	−1.82
13	dbdppz	XantPhos	50	408	13754	75	565	2.19	−1.29
14	dbdppz	dppf	79	413	11711	69	597	2.08	−0.80
15	bdppz	BINAP	53	462	5930	2.3	560	2.21	−1.20

*^a^* Isolated yields following precipitation with Et_2_O; * The astericks demotes the excited state of Cu(I).

## Data Availability

Samples of the compounds are not available from the authors.

## Supplementary Materials

The following supporting information can be downloaded at: https://www.mdpi.com/article/10.3390/molecules27123745/s1, experimental procedures, photophysical data and NMR and mass spectra [37,38,39,40,41].
