# Supplementary material for: Heteroleptic Copper(I)-Based Complexes Incorporating BINAP and π-Extended Diimines: Synthesis, Catalysis and Biological Applications
Source: Molecules. 2022 Jun 10;27(12):3745. doi: 10.3390/molecules27123745 (PMC9230762; doi:10.3390/molecules27123745)
Supplement: Supplementary file 1 [file molecules-27-03745-s001.zip › molecules-1747788-supplementary.pdf]

SUPPORTING INFORMATION

Heteroleptic Copper(I)-Based Complexes Incorporating BINAP and π-Extended Diimines: Synthesis, Catalysis and Biological Applications.

Corentin Cruché, Sayak Gupta,[†] Jeremy Kodanko*[†] and Shawn K. Collins*

Département de Chimie, Centre for Green Chemistry and Catalysis, Université de Montréal, 1375 Avenue Thérèse-Lavoie-Roux, Montréal, QC H2V 0B3 CANADA. [†] Department of Chemistry, Wayne State University, 5101 Cass Ave, Detroit, MI 48202, USA. jkodanko@wayne.edu, shawn.collins@umontreal.ca

Table of Contents

Table of Contents 1
GENERAL : 1
SYNTHESIS OF LIGANDS AND CATALYSTS 2
General Comments/Procedures for Ligands: 2
Synthesis of Copper Complexes 3
General Procedure for the Synthesis of Heteroleptic Complexes: 3
SUBSTRATE SYNTHESIS 5
PHOTOPHYSICAL DATA 7
Absorbance Spectra 7
Emission Spectra 7
EXCITED STATE LIFETIME DATA 9
Electrochemistry 11
Experimental Procedure for Biological Testing 12
NMR Spectra 13
Ligands 13
Copper Complexes 15

GENERAL :

All reactions that were carried out under anhydrous conditions were performed under an inert argon or nitrogen atmosphere in glassware that had previously been dried overnight at 120 °C or had been flame dried and cooled under a stream of argon or nitrogen. All chemical products were obtained from Sigma-Aldrich Chemical Company, Oakwood Chemical or Alfa Aesar and were reagent quality. Technical solvents were obtained from VWR International Co. Anhydrous solvents (CH_2Cl_2, Et_2O, THF, DMF, toluene, and *n*-hexane) were dried and deoxygenated using a GlassContour system (Irvine, CA). Isolated yields reflect the mass obtained following flash column silica gel chromatography. Organic compounds were purified using silica gel obtained from Silicycle Chemical division (40-63 nm; 230-240 mesh). Analytical thin-layer chromatography (TLC) was performed on glass-backed silica gel 60 coated with a fluorescence indicator (Silicycle Chemical division, 0.25 mm, F254.).

Visualization of TLC plate was performed by UV (254 nm), $KMnO_4$ or *p*-anisaldehyde stains. All mixed solvent eluents are reported as v/v solutions. Concentration refers to removal of volatiles at low pressure on a rotary evaporator. All reported compounds were homogeneous by thin layer chromatography (TLC) and by ^1H NMR. NMR spectra were taken in deuterated $CDCl_3$ using Bruker AV-300 and AV-400 instruments unless otherwise noted. Signals due to the solvent served as the internal standard ($CHCl_3$: δ 7.27 for 1H, δ 77.0 for 13C). The acquisition parameters are shown on all spectra. The ^1H NMR chemical shifts and coupling constants were determined assuming first-order behavior. Multiplicity is indicated by one or more of the following: s (singlet), d (doublet), t (triplet), q (quartet), m (multiplet), br (broad); the list of couplings constants (*J*) corresponds to the order of the multiplicity assignment. High resolution mass spectroscopy (HRMS) was done by the Centre régional de spectrométrie de masse at the Département de Chimie, Université de Montréal from an Agilent LC-MSD TOF system using ESI mode of ionization unless otherwise noted.

SYNTHESIS OF LIGANDS AND CATALYSTS

General Comments/Procedures for Ligands:

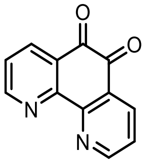

1,10-Phenanthroline-5,6-dione: 1,10-Phenanthroline (3.00 g, 16.6 mmol) was dissolved in concentrated H_2SO_4 (20 mL) and H_2O (10 mL) at 0°C. $NaBrO_3$ (2.76 g, 18.3 mmol, 1.1 eq.) in H_2O (10 mL) was then added dropwise. The yellow solution was then stirred for 36 h. The mixture was then neutralized under intense stirring, using a saturated solution of K_2CO_3. The mixture was extracted with CH_2Cl_2 three times, and the organic phase dried with $MgSO_4$. The solution was concentrated *in vacuo*. Purification by flash column chromatography (100% CH_2Cl_2 → 95:5 CH_2Cl_2:MeOH) afforded the pure product as a yellow powder (1.89 g, 54%). Spectral data were in accordance with previous reports [37]. **^1H NMR (500 MHz, $CDCl_3$):** δ 9.12 (dd, *J* = 4.7, 1.9 Hz, 2H), 8.51 (dd, *J* = 7.9, 1.8 Hz, 2H), 7.59 (dd, *J* = 7.9, 4.7 Hz, 2H).

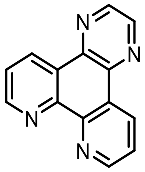

Pyrazino[2,3-f][1,10]phenanthroline (dpq): 1,10-Phenanthroline-5,6-dione (400 mg, 1.9 mmol, 1.0 equiv), ethylenediamine (152 µL, 2.28 mmol, 1.2 equiv.) and *p*-toluenesulfonic acid (5.78 mg, 0.02 eq.) were all dissolved in EtOH (10 mL, 0.19 M). The solution was then refluxed for 18 h in a sealed tube. The solution was cooled down to room temperature and water was added. The mixture was extracted with CH_2Cl_2 three times, and the organic phase dried with $MgSO_4$. The solution was concentrated *in vacuo*. Purification by flash column chromatography (100% CH_2Cl_2 → 95:5 CH_2Cl_2:MeOH) afforded the pure product as a pale orange solid (365 mg, 83 %). Spectral data were in accordance with previous reports [38]. **^1H NMR (500 MHz, $CDCl_3$):** δ 9.52 (dd, *J* = 8.1, 1.8 Hz, 2H), 9.32 (dd, *J* = 4.4, 1.8 Hz, 1H), 9.00 (s, 2H), 7.82 (dd, *J* = 8.1, 4.4 Hz, 2H).

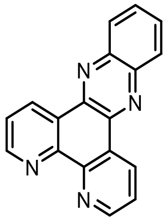

Dipyrido[3,2-a:2',3'-c]phenazine (dppz): 1,10-Phenanthroline-5,6-dione (500 mg, 2.38 mmol, 1.0 eq.), 1,2-phenylenediamine (289mg, 2.62 mmol, 1.1 eq.) and *p*-toluenesulfonic acid (4.52 mg, 28.8 µmol, 0.01 eq.)

were all dissolved in EtOH (10 mL, 0.19 M). The solution was then refluxed for 18 h in a sealed tube. The solution was cooled down to room temperature and water was added. The mixture was extracted with CH_2Cl_2 three times, and the organic phase dried with $MgSO_4$. The solution was concentrated *in vacuo*. Purification by flash column chromatography (100% CH_2Cl_2 → 95:5 CH_2Cl_2:MeOH) afforded the pure product as an orange solid (432 mg, 64 %). Spectral data were in accordance with previous reports [39]. **^{1}H NMR (500 MHz, $CDCl_3$):** δ 9.67 (dd, *J* = 8.2, 2.0 Hz, 2H), 9.32 (s, 2H), 8.39 – 8.32 (m, 2H), 7.97 – 7.90 (m, 1H), 7.87 – 7.80 (m, 2H).

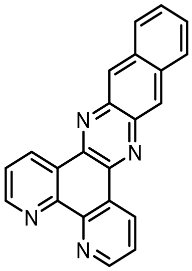

Benzo[i]dipyrido[3,2-a:2',3'-c]phenazine (bdppz): 1,10-Phenanthroline-5,6-dione (200 mg, 0.95 mmol, 1 eq.), 2,3-Diaminonaphthalene (166 mg, 1.05 mmol, 1.1 eq.) and *p*-toluenesulfonic acid (1.81 mg, 9.52 µmol, 0.01 eq.) were all dissolved in EtOH (10 mL, 0.19 M). The solution was then refluxed in a sealed tube for 30 min until a precipitate formed. The mixture was cooled down to room temperature, the solid was filtered and washed with a minimum of acetone to give the pure product as a dark orange solid (134 mg, 42 %). Spectral data were in accordance with previous reports [39]. **^{1}H NMR (500 MHz, $CDCl_3$):** δ 9.70 (d, *J* = 8.1 Hz, 2H), 9.41 – 9.25 (m, 2H), 8.96 (s, 2H), 8.21 (dd, *J* = 6.4, 3.3 Hz, 2H), 7.93 – 7.78 (m, 2H), 7.65 (dd, *J* = 6.6, 3.1 Hz, 2H).

Synthesis of Copper Complexes

General Procedure for the Synthesis of Heteroleptic Complexes:

To a stirred solution of $[Cu(MeCN)_4]BF_4$ (1.0 equiv.) in anhydrous CH_2Cl_2 (0.032 M) was added the corresponding phosphine (1.05 equiv.). The reaction was stirred at room temperature for one hour. Then, to the reaction mixture was added the corresponding diimine (1.05 equiv.). The reaction mixture was stirred for an additional hour. The reaction mixture was concentrated to approximately a fifth of the original volume and Et_2O was added dropwise to precipitate the product. Filtration afforded the desired complex as a coloured solid.

Note: Complexes bearing S- or R-BINAP were synthetized according to the same procedure and the corresponding ^{1}H NMR were in accordance with the racemic analogs.

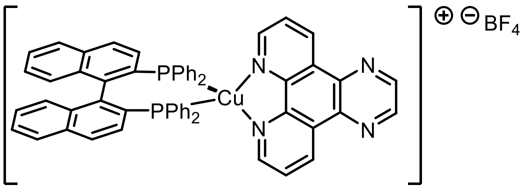

[Cu(dpq)(BINAP)]BF_4 : Following the general procedure, $[Cu(MeCN)_4]BF_4$ (50.0 mg, 0.16 mmol, 1 equiv.) and (rac)-2,2-bis(diphenylphosphino)-1,1-binaphthyl (99.2 mg, 0.16 mmol, 1 eq.) were dissolved in anhydrous dichloromethane (25 mL). After one hour of mixing, dpq (37.0 mg, 0.16 mmol, 1 eq.) was added. The reaction mixture was stirred for another hour. The solution was concentrated *in vacuo,* and 30 mL of diethyl ether was added to the solution. The desired product was obtained by filtration as a yellow solid (120 mg, 75%). **^{1}H NMR (700 MHz, Acetone-d_6):** δ 9.83 (dd, *J* = 8.2, 1.5 Hz, 2H), 9.38 – 9.35 (m, 2H), 9.31 (s, 2H), 8.31

(dd, *J* = 8.1, 4.8 Hz, 2H), 7.93 (d, *J* = 8.6 Hz, 2H), 7.80 (d, *J* = 1.2 Hz, 1H), 7.79 (s, 1H), 7.53 – 7.44 (m, 8H), 7.43 (s, 1H), 7.36 – 7.32 (m, 2H), 7.33 – 7.27 (m, 4H), 7.27 – 7.22 (m, 6H), 7.22 (s, 2H), 6.97 (d, *J* = 8.6 Hz, 2H), 6.89 – 6.85 (m, 2H), 6.74 – 6.70 (m, 4H). **^{13}C NMR (176 MHz, Acetone-d$_6$):** δ 153.3, 147.3, 145.8, 140.7, 140.3, 135.7, 135.2, 134.7, 134.3, 134.0, 133.5, 132.3, 131.5, 130.3, 130.3, 130.0, 130.0, 130.0, 129.0, 128.5, 128.3, 128.2, 127.8, 127.5, 127.4. **HRMS (ESI)** m/z calculated for $C_{58}H_{40}CuN_4P_2$ $[M]^+$ 917.2019; found 917.2006; **HRMS (ESI)** m/z calculated for $[^{11}B]F_4$ $[M]^-$ 87.0029; found 87.0048.

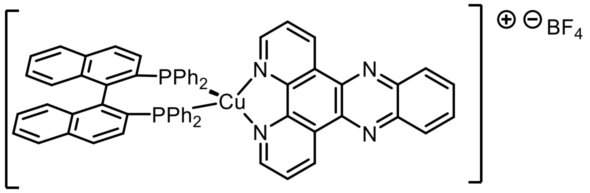

[Cu(dppz)(BINAP)]BF$_4$: Following the general procedure, $[Cu(MeCN)_4]BF_4$ (50.0 mg, 0.16 mmol, 1 equiv.) and (rac)-2,2-bis(diphenylphosphino)-1,1-binaphthyl (99.2 mg, 0.16 mmol, 1 eq.) were dissolved in anhydrous dichloromethane (25 mL). After one hour of mixing, dppz (45.0 mg, 0.16 mmol, 1 eq.) was added. The reaction mixture was stirred for another hour. The solution was concentrated *in vacuo,* and 30 mL of diethyl ether was added to the solution. The desired product was obtained by filtration as a brown solid (132 mg, 77**%). ^{1}H NMR (700 MHz, Acetone-d$_6$)**: δ 9.93 (dd, *J* = 8.0, 1.5 Hz, 2H), 9.34 (dd, *J* = 4.9, 1.4 Hz, 2H), 8.49 (dd, *J* = 6.4, 3.4 Hz, 2H), 8.31 (dd, *J* = 8.0, 4.8 Hz, 2H), 8.20 – 8.14 (m, 2H), 7.94 (d, *J* = 8.6 Hz, 2H), 7.81 (dd, *J* = 8.3, 1.3 Hz, 2H), 7.56 – 7.51 (m, 4H), 7.53 – 7.44 (m, 4H), 7.38 – 7.31 (m, 4H), 7.34 – 7.30 (m, 2H), 7.30 – 7.23 (m, 6H), 6.98 (dd, *J* = 8.6, 1.1 Hz, 2H), 6.91 – 6.85 (m, 2H), 6.74 (dd, *J* = 7.4 Hz, 4H). **^{13}C NMR (176 MHz, Acetone-d$_6$):** δ 153.4, 146.8, 143.6, 141.2, 140.4, 136.1, 135.2, 134.8, 134.3, 134.0, 133.5, 133.0, 132.3, 131.5, 130.5, 130.5, 130.4, 130.3, 130.1, 130.0, 129.0, 128.5, 128.3, 128.2, 127.9, 127.6, 127.6; **HRMS (ESI)** m/z calculated for $C_{62}H_{42}CuN_4P_2$ $[M]^+$ 967.2175; found 967.2176; **HRMS (ESI)** m/z calculated for $[^{11}B]F_4$ $[M]^-$ 87.0029; found 87.0050.

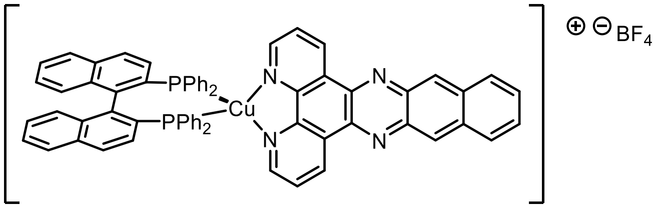

[Cu(bdppz)(BINAP)]BF$_4$: Following the general procedure, $[Cu(MeCN)_4]BF_4$ (50.0 mg, 0.16 mmol, 1 equiv.) and (rac)-2,2-bis(diphenylphosphino)-1,1-binaphthyl (99.2 mg, 0.16 mmol, 1 eq.) were dissolved in anhydrous dichloromethane (25 mL). After one hour of mixing, bdppz (52.9 mg, 0.16 mmol, 1 eq.) was added. The reaction mixture was stirred for another hour. The solution was concentrated *in vacuo,* and 30 mL of diethyl ether was added to the solution. The desired product was obtained by filtration as a brown solid (93 mg, 53%). **^{1}H NMR (700 MHz, Acetone-d$_6$):** δ 9.84 (d, *J* = 7.9 Hz, 2H), 9.32 (d, *J* = 4.7 Hz, 2H), 9.05 (s, 2H), 8.31 – 8.25 (m, 4H), 7.94 (d, *J* = 8.5 Hz, 2H), 7.81 (d, *J* = 8.3 Hz, 2H), 7.65 (d, *J* = 7.3 Hz, 2H), 7.56 (q, *J* = 6.1 Hz, 4H), 7.48 (dd, *J* = 7.4 Hz, 5H), 7.34 (td, *J* = 16.8, 15.1, 8.8 Hz, 8H), 7.31 – 7.25 (m, 4H), 7.24 (d, *J* = 7.4 Hz, 2H), 6.98 (d, *J* = 8.5 Hz, 2H), 6.89 (dd, *J* = 7.4 Hz, 2H), 6.75 (dd, *J* = 7.5 Hz, 4H). **^{13}C NMR (176 MHz, Acetone-d$_6$):** δ 153.50, 147.27, 142.06, 140.35, 139.47, 136.12, 135.80, 135.27, 134.75, 134.33, 133.99, 133.50, 132.29, 131.51, 130.78, 130.39, 130.27, 130.09, 130.07, 129.42, 129.04, 128.93, 128.67, 128.53, 128.34, 128.15, 127.86, 127.76, 127.55. **HRMS (ESI)** m/z calculated for $C_{66}H_{44}CuN_4P_2$ $[M]^+$ 1017.23317; found 1017.23595; **HRMS (ESI)** m/z calculated for $[^{11}B]F_4$ $[M]^-$ 87.0035; found 87.0047.

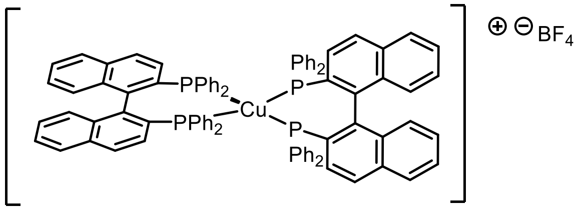

[Cu(BINAP)$_2$]BF$_4$: [Cu(MeCN)$_4$]BF$_4$ (50.0 mg, 0.16 mmol, 1 equiv.) and (rac)-2,2-Bis(diphenylphosphino)-1,1-binaphthyl (198 mg, 0.32 mmol, 2 eq.) were dissolved in anhydrous dichloromethane (5 mL) in a sealed tube. The solution was sparged with N_2 for 5 min, and the reaction was mixed at 90°C for 16h. The reaction was cooled down, and 40 mL of deoxygenated Et_2O was added. The desired product was obtained by filtration as a white powder (183 mg, 82%). **^{1}H NMR (500 MHz, Acetone-d$_6$)**: δ 8.02 (d, *J* = 6.9 Hz, 8H), 7.95 (d, *J* = 8.8 Hz, 4H), 7.74 – 7.70 (m, 4H), 7.68 – 7.63 (m, 9H), 7.53 (dd, *J* = 7.6 Hz, 8H), 7.46 (ddd, *J* = 8.0, 6.8, 1.1 Hz, 4H), 7.27 – 7.21 (m, 12H), 7.06 (dd, *J* = 8.7, 1.0 Hz, 4H), 6.47 (dd, *J* = 7.4 Hz, 4H), 5.96 (dd, *J* = 7.6 Hz, 8H).**^{13}C NMR (176 MHz, Acetone-d$_6$):** δ 140.74, 137.86, 134.83, 134.31, 134.11, 132.91, 132.44, 132.03, 129.76, 129.68, 129.61, 129.36, 129.01, 128.09, 127.80, 127.53, 127.32. **HRMS (ESI)** m/z calculated for $C_{88}H_{64}CuP_4$ [M]$^+$ 1307.32490; found 1307.33098; **HRMS (ESI)** m/z calculated for [^{11}B]F$_4$ [M]$^-$ 87.0035; found 87.0042.

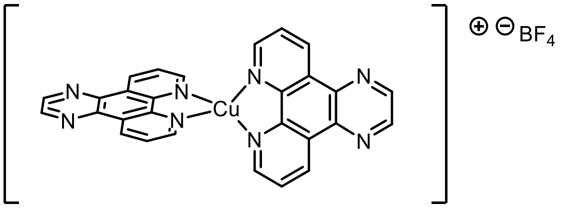

[Cu(dpq)$_2$]BF$_4$: Following the general procedure, [Cu(MeCN)$_4$]BF$_4$ (50.0 mg, 0.16 mmol, 1 equiv.) and dpq (74 mg, 0.38 mmol, 2 eq.) were dissolved in anhydrous dichloromethane (25 mL). After one hour of mixing, the solution was concentrated *in vacuo,* and 30 mL of diethyl ether was added to the solution. The desired product was obtained by filtration as a brown solid (74.2 mg, 76%). Note that we observed broad peaks in the NMR spectra as reported by others who prepared the complex through a different route [40]. **^{1}H NMR (500 MHz, DMSO-d$_6$):** δ 9.81 – 9.55 (m, 1H), 9.43 – 9.27 (m, 1H), 9.26 – 9.08 (m, 1H), 8.40 – 8.09 (m, 1H). **HRMS (ESI)** m/z calculated for $C_{28}H_{16}CuN_8$ [M]$^+$ 527.0788; found 527.0798; **HRMS (ESI)** m/z calculated for [^{11}B]F$_4$ [M]$^-$ 87.0035; found 87.0041.

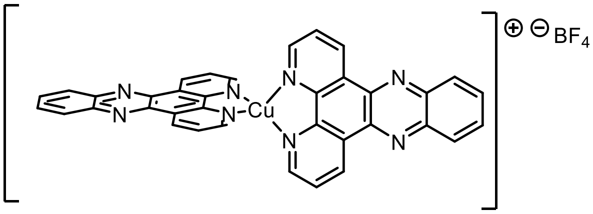

[Cu(dppz)$_2$]BF$_4$: : Following the general procedure, [Cu(MeCN)$_4$]BF$_4$ (50.0 mg, 0.16 mmol, 1 equiv.) and dpq (89 mg, 0.38 mmol, 2 eq.) were dissolved in anhydrous dichloromethane (25 mL). After one hour of mixing, the solution was concentrated *in vacuo,* and 30 mL of diethyl ether was added to the solution. The desired product was obtained by filtration as a brown solid (83.9 mg, 74%). *Note: Similar NMR spectra as [Cu(dpq)$_2$]BF$_4$ was observed.* ^{1}H NMR (500 MHz, DMSO) δ 9.92 – 9.68 (m, 1H), 9.30 – 9.13 (m, 1H), 8.62 – 8.42 (m, 1H), 8.36 – 8.09 (m, 2H). **HRMS (ESI)** m/z calculated for $C_{36}H_{20}CuN_8$ [M]$^+$ 627.1101; found 627.1114; **HRMS (ESI)** m/z calculated for [^{11}B]F$_4$ [M]$^-$ 87.0051; found 87.0035.

SUBSTRATE SYNTHESIS

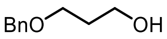

3-(Benzyloxy)propanol : The benzyl(Bn)-protected alcohol was prepared using a procedure reported by Gilmore et al. [41]. NMR data was in accordance with what was previously reported.

General Procedure for the Appel Reaction: To an open oven-dried reaction vial charged with a stir bar was added the copper catalyst (0.002 mmol, 0.01 equiv), the alcohol (33 mg, 0.20 mmol, 1.0 equiv), carbon tetrabromide (67 mg, 0.4 mmol, 2.0 equiv) and sodium bromide (41 mg, 0.40 mmol, 2.0 equiv). The flask vial was capped, purged with a stream of nitrogen and dry DMF (1.5 mL) was added via syringe. The reaction mixture was stirred

under 400 nm irradiation for 24 h. The mixture was poured into a separatory funnel containing Et_2O (10 mL) and H_2O (10 mL). The layers were separated, and the aqueous layer was extracted with Et_2O (2 × 10 mL). The combined organic layers were washed with sat. $Na_2S_2O_3$ solution, brine, dried over Na_2SO_4 and concentrated *in vacuo*. The residue was purified by chromatography (100 % Hexanes) to afford the desired product as a colorless oil. NMR data was in accordance with what was previously reported [26].

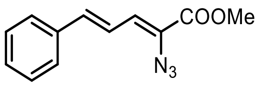

Methyl (2Z,4E)-2-azido-5-phenylpenta-2,4-dienoate: To a cooled (–22 °C) solution of NaOMe (2.67 mL, 9.93 mmol, 2.5 equiv.) in MeOH (2 mL) was added a solution of cinnamaldehyde (0.50 mL, 3.97 mmol, 1 equiv.) and methyl azidoacetate (9.93 mmol, 0.97 mL, 2.5 equiv.) dropwise over 20 min. The resulting reaction mixture was warmed to 10 °C. After four hours, the heterogeneous mixture was diluted with water (10 mL) and EtOAc (10 mL). The phases were separated, and the resulting aqueous phase was extracted with additional EtOAc (2 × 10 mL). The combined organic phases were washed with distilled water (2 × 10 mL) and brine (10 mL). The combined organic phases were dried over Na_2SO_4, filtered and the filtrate was concentrated in vacuo. The crude residue was purified by flash column chromatography (0:100 – 5:95 EtOAc:hexanes) to afforded the product as a colorless oil (628 mg, 69 %). NMR data was in accordance with what was previously reported [33].

General Procedure for the Sensitization of Vinyl Azides: To an oven-dried 4 mL vial with a stir bar were added the azide (46 mg, 0.20 mmol, 1 equiv.), the copper catalyst (0.002 mmol, 0.01 equiv.) and dry and degassed $CHCl_3$ (2 mL, 0.1 M). The solution was purged with N_2 and irradiated at room temperature with a 1W blue light-emitting diode (LED) strip for 3 hours. The mixture was concentrated in vacuo, and the crude residue was purified by silica gel flash column chromatography (100% CH_2Cl_2) to afford the desired pyrrole as a white solid. NMR data was in accordance with what was previously reported [42].

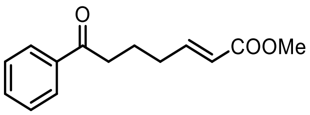

Methyl (E)-7-oxo-7-phenylhept-2-enoate: The ketone was prepared using a modified procedure reported by Knowles. Spectroscopic data was in accordance with what was previously reported [30].

General Procedure for the PCET Reaction: To an oven dried reaction vial charged with a stir bar is added (35.6 mg, 0.16 mmol, 1 eq.), the copper catalyst (0.00314 mmol, 5 mol %), Hantzsch ester (55.34mg, 0.24 mmol 1.5 eq.), diphenylphosphoric acid (2mg, 0.00785 mmol 5 mol %), and THF (3.14 ml 0.05M). The mixture is then degassed for 5 minutes with a nitrogen stream and irradiated over night. The reaction mixture is then directly purified by flash chromatography (100 % →10%, *n*-hexanes:EtOAc). The yield of the desired bicyclic lactone is calculated by NMR. Spectroscopic data was in accordance with what was previously reported [26].

PHOTOPHYSICAL DATA

Table S1. Tabular data for the maximum of absorbance and emission of copper complexes

Cu(**NN**)(BINAP)BF_4	λ_{abs} (nm)	E (L/mol cm)	λ_{em} (nm)	E_T (eV)
DPQ	424	5752	521	2.38
DPPZ	433	6759	545	2.27
BDPPZ	462	5930	560	2.21

Absorbance Spectra

Absorbance UV-Vis spectra were recorded with Varian Cary 5000 UV-Vis-NIR spectrophotometer in a quartz cuvette.

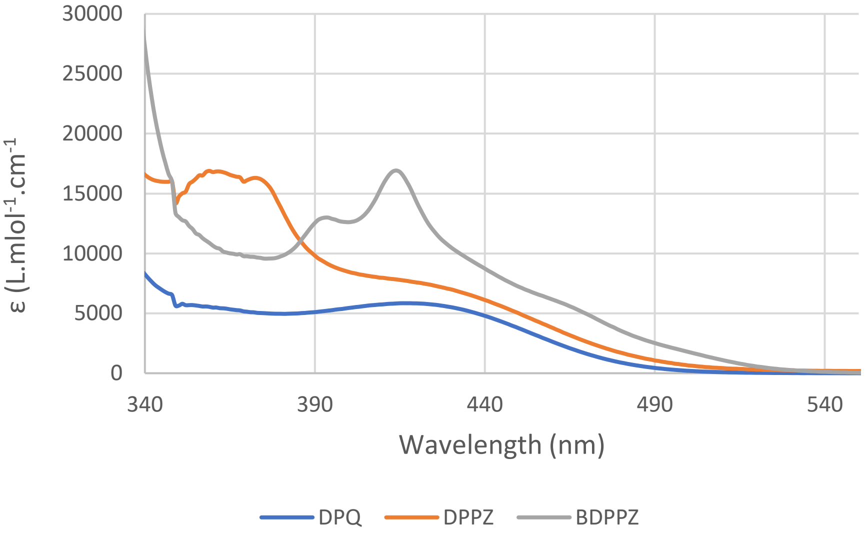

Figure S2. UV-visible absorption spectrum of new Cu(**NN**)(**BINAP**)BF_4 complexes recorded at ambient temperature in CH_2Cl_2 ($1.0\cdot10^{-4}$M)

Emission Spectra

Emission spectra were recorded with the Varian Cary Eclipse Fluorescence Spectrophotometer/Fluorometer in a quartz cuvette.

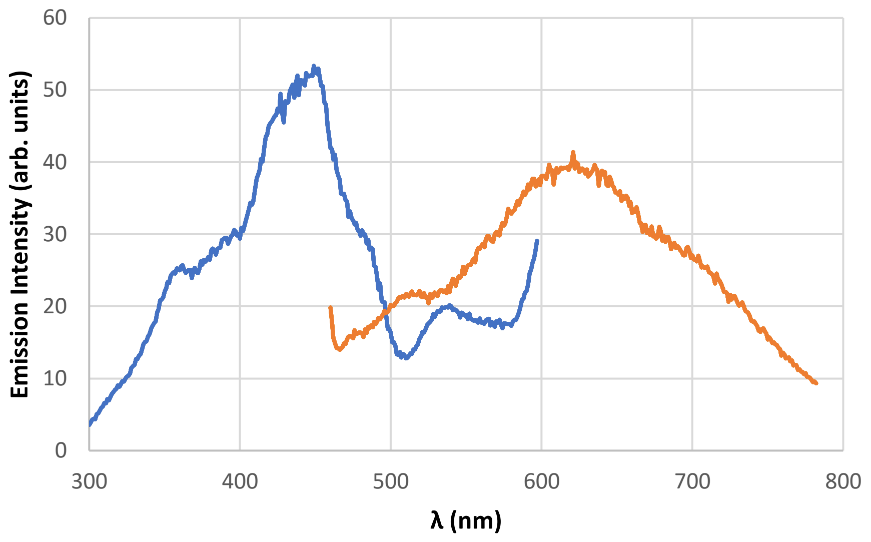

Figure S3. Excitation (blue curve) and emission (orange curve) spectrum of Cu(dpq)(BINAP)BF_4 excited at 450 nm, recorded at ambient temperature in CH_2Cl_2 ($5.0 \cdot 10^{-4}$M).

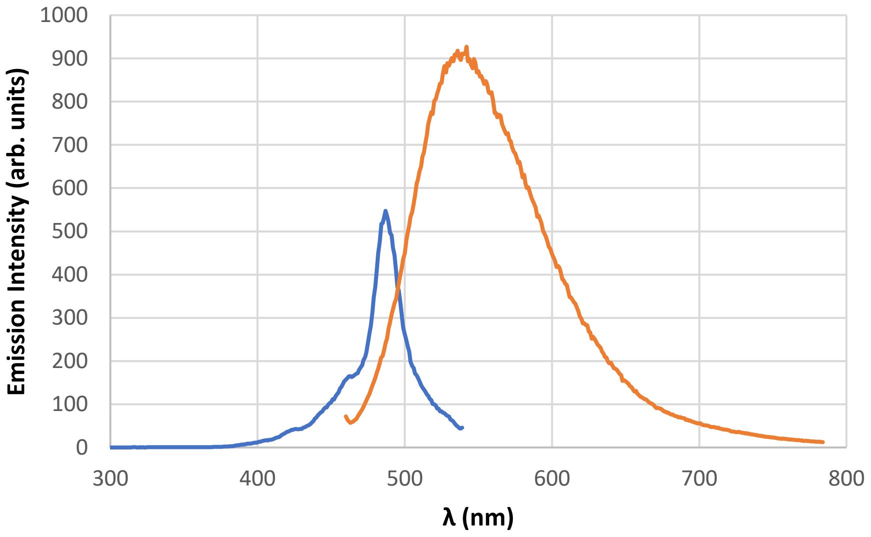

Figure S4. Excitation (blue curve) and emission (orange curve) spectrum of Cu(dppz)(BINAP)BF_4 excited at 450 nm, recorded at ambient temperature in CH_2Cl_2 ($5.0 \cdot 10^{-4}$M).

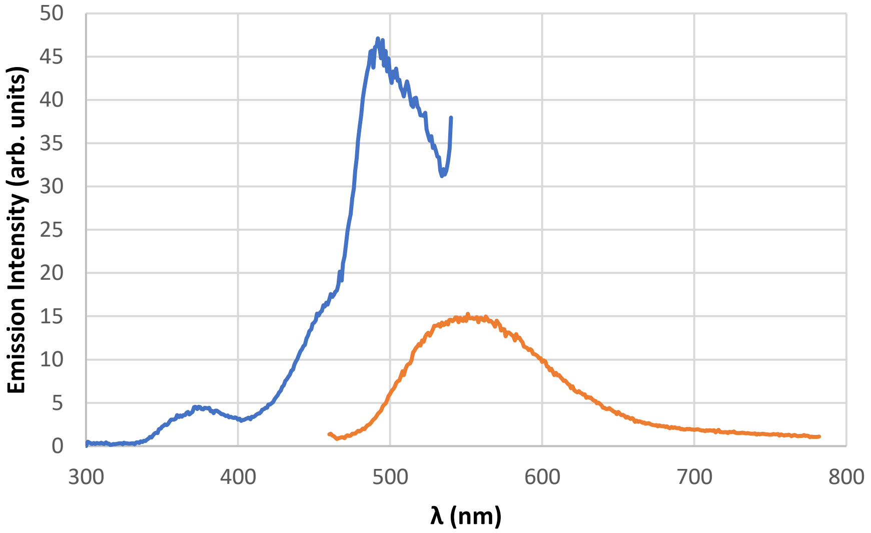

Figure S5. Excitation (blue curve) and emission (orange curve) spectrum of Cu(bdppz)(BINAP)BF_4 excited at 450 nm, recorded at ambient temperature in CH_2Cl_2 ($5.0 \cdot 10^{-4}$M).

EXCITED STATE LIFETIME DATA

Lifetime measurements were done with an Edinburgh Instruments Mini Tau Lifetime Fluorimeter with an EPL 405 laser (exciting at 405 nm) with a standard deviation of 1-2%.

Table S6. Tabular data for the excited state lifetime of copper complexes

Cu(**NN**)(BINAP)BF_4	τ (ns)
dpq	1.4
dppz	1.8
bdppz	2.3

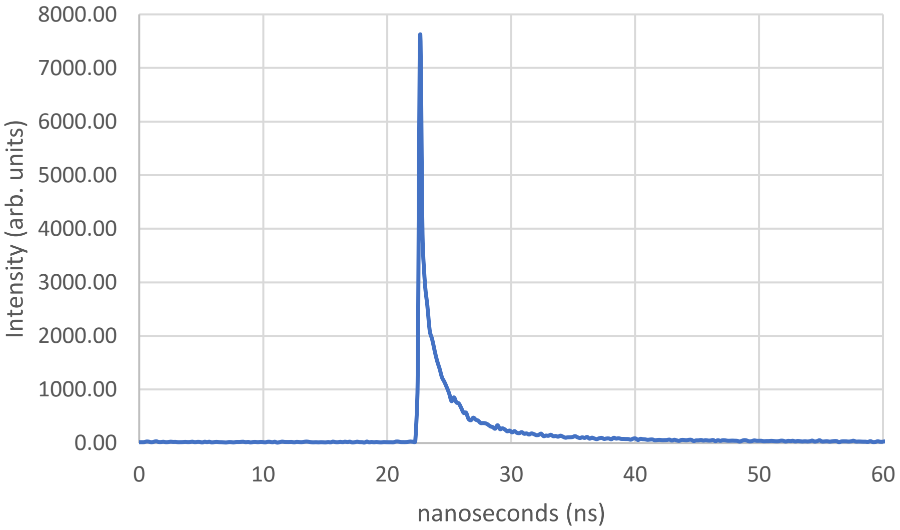

Figure S7. Lifetime spectrum of Cu(dpq)(BINAP)BF_4 excited at 405 nm, recorded at ambient temperature in degassed CH_2Cl_2 ($1.25. 10^{-4}$ M).

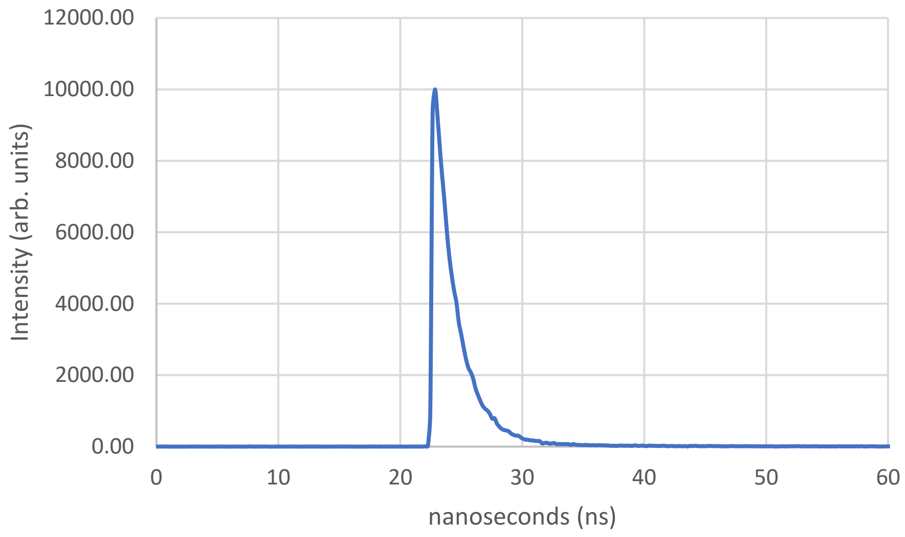

Figure S8. Lifetime spectrum of Cu(dppz)(BINAP)BF_4 excited at 405 nm, recorded at ambient temperature in degassed CH_2Cl_2 (1.25. 10^{-4} M).

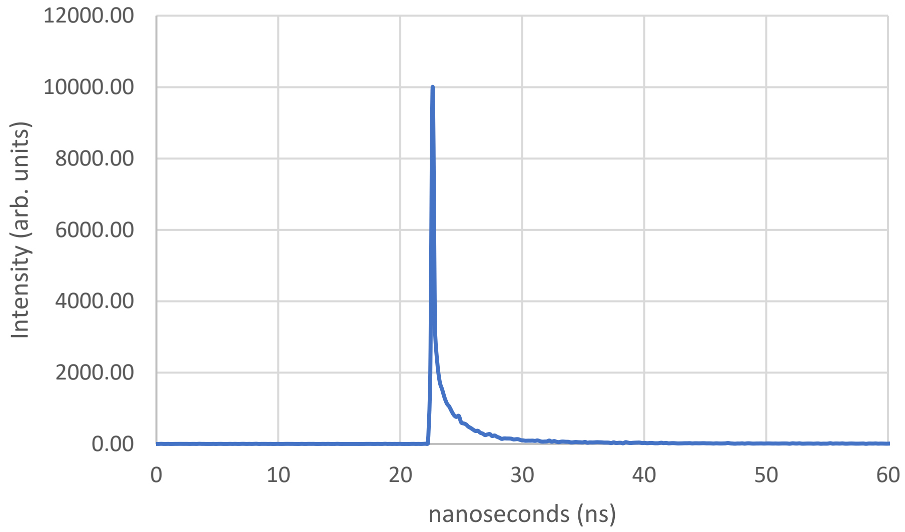

Figure S9. Lifetime spectrum of Cu(bdppz)(BINAP)BF_4 excited at 405 nm, recorded at ambient temperature in degassed CH_2Cl_2 (1.25. 10^{-4} M).

Electrochemistry

Osteryoung Square Wave Voltammetry (OSWV) measurements were done in dry acetonitrile (MeCN) with tetra-*N*-butylammonium hexafluorophosphate ($TBAPF_6$) as the supporting electrolyte using a standard three-electrode cell consisting of a silver wire pseudo-reference electrode, a platinum wire counter electrode and a platinum working electrode with a BioLogic SP-50 potentiostat. The solutions were degassed with a flow of argon for 5 minutes prior to the measurements, which was left over the surface of the solution during the measurements. All measurements were carried out at 22 °C. All potentials were reported to the potential of the ferrocene/ferrocenium (Fc/Fc+) redox couple which was determined in the aforementioned electrolyte.

Cu(**NN**)(BINAP)BF_4	E_{ox} (V)	E_{red} (V)	ΔE (V)
dpq	0.98	-1.14	2.12
dppz	1.01	-0.95	1.96
bdppz	1.01	-0.89	1.90

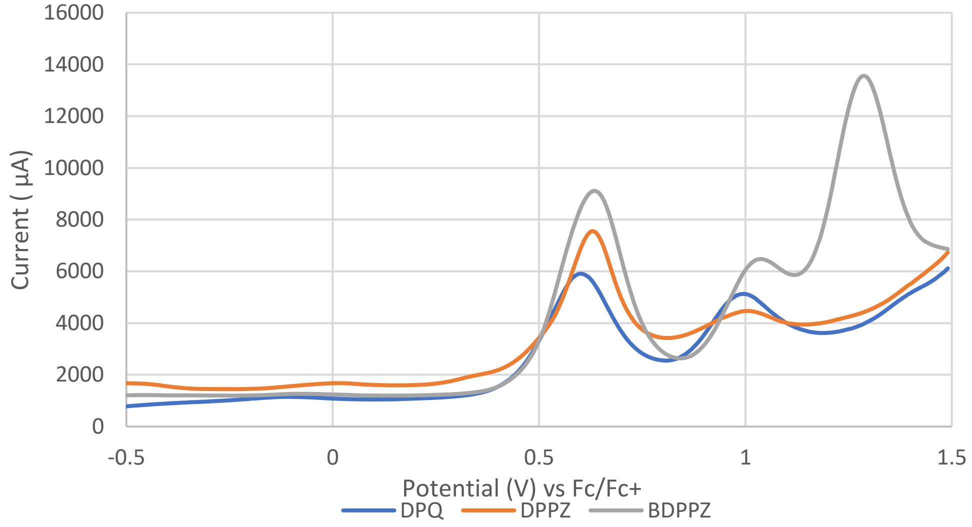

Figure S10. OSWVs of Cu(**NN**)(BINAP)BF_4 in MeCN (1 mM) using $TBAPF_6$ (100 mM) as supporting electrolyte (Anodic scan, frequency 20 Hz, amplitude 20 mV, step potential 5 mV).

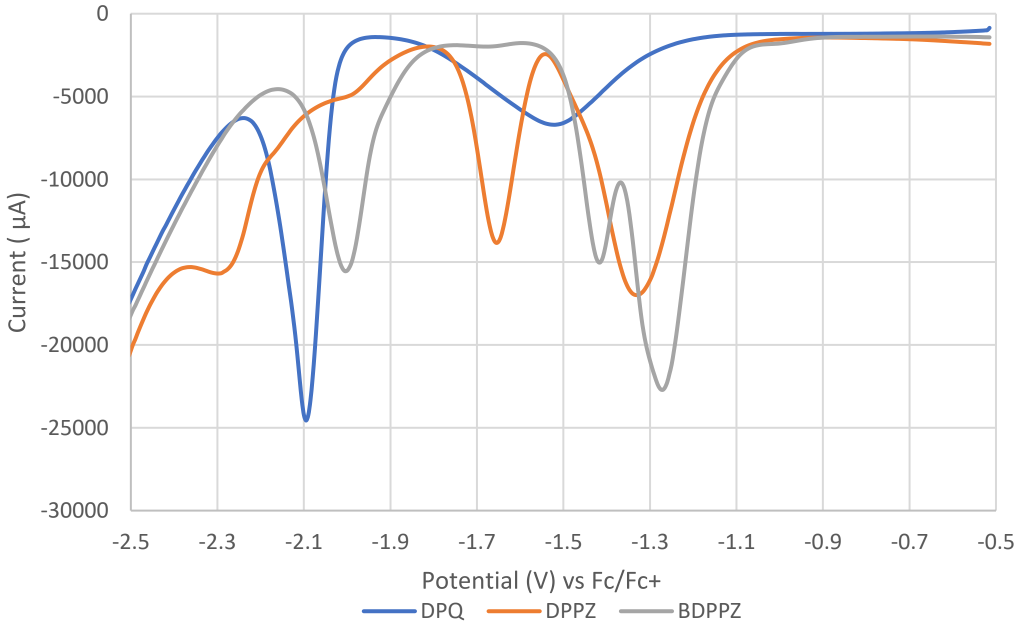

Figure S11 OSWVs of Cu(**NN**)(BINAP)BF_4 in MeCN (1 mM) using $TBAPF_6$ (100 mM) as supporting electrolyte (Cathodic scan, frequency 20 Hz, amplitude 20 mV, step potential 5 mV).

Experimental Procedure for Biological Testing

The biological activity of all the synthesized complexes **1-9** and controls **10-12** were determined by plating MDA-MB-231 in a 96 well plate at a density of 7,000 cells per well in MDA-MB-231 cell growth medium (Dulbecco's Modified Eagle's Medium (DMEM) supplemented with 10% FBS and 1,000 units/mL penicillin/streptomycin). The plates were incubated overnight in a 37°C humidified incubator ventilated with 5% CO_2. The growth media was aspirated off and then quadruplicate wells were treated with growth media containing 5 µM of the synthesized complexes or controls in 1% DMSO. The plate also contained wells with no cells which were designated as blank wells whereas wells with cells that were not treated with the compound but only with growth media containing 1% DMSO (vehicle) were designated as control wells. Following the addition of 5 µM of the synthesized complexes or controls, the plates were incubated in a 37°C humidified incubator ventilated with 5% CO_2 for 72 h. After incubation, 10 µL of 3-(4,5-dimethylthiazol-2-yl)-2,5-diphenyl tetrazolium bromide (MTT) reagent (5 mg/mL in PBS) was added to each well of the 96 well plate and incubated in a 37°C humidified incubator ventilated with 5% CO_2 for 2 h. After 2 h the media was aspirated off and 100 µL of DMSO was added. The plates were then shaken for 20 min to ensure complete dissolution of the purple formazan crystals formed. Absorbance of each well was then measured at 570 nm. The mean absorbance values of the blank wells were calculated and subtracted from absorbance values for each well treated with 5 µM of the synthesized complex or controls. The absorbance of the control wells was also taken and subtracted with the average of the blank wells. The mean of these corrected control absorbances were then calculated. Viability of the cells was finally determined by dividing the corrected absorbance of the wells containing 5 µM of the synthesized complexes or controls by the mean corrected absorbance of the control wells and expressing the mean of the ratio as a percentage value.

NMR Spectra

Ligands

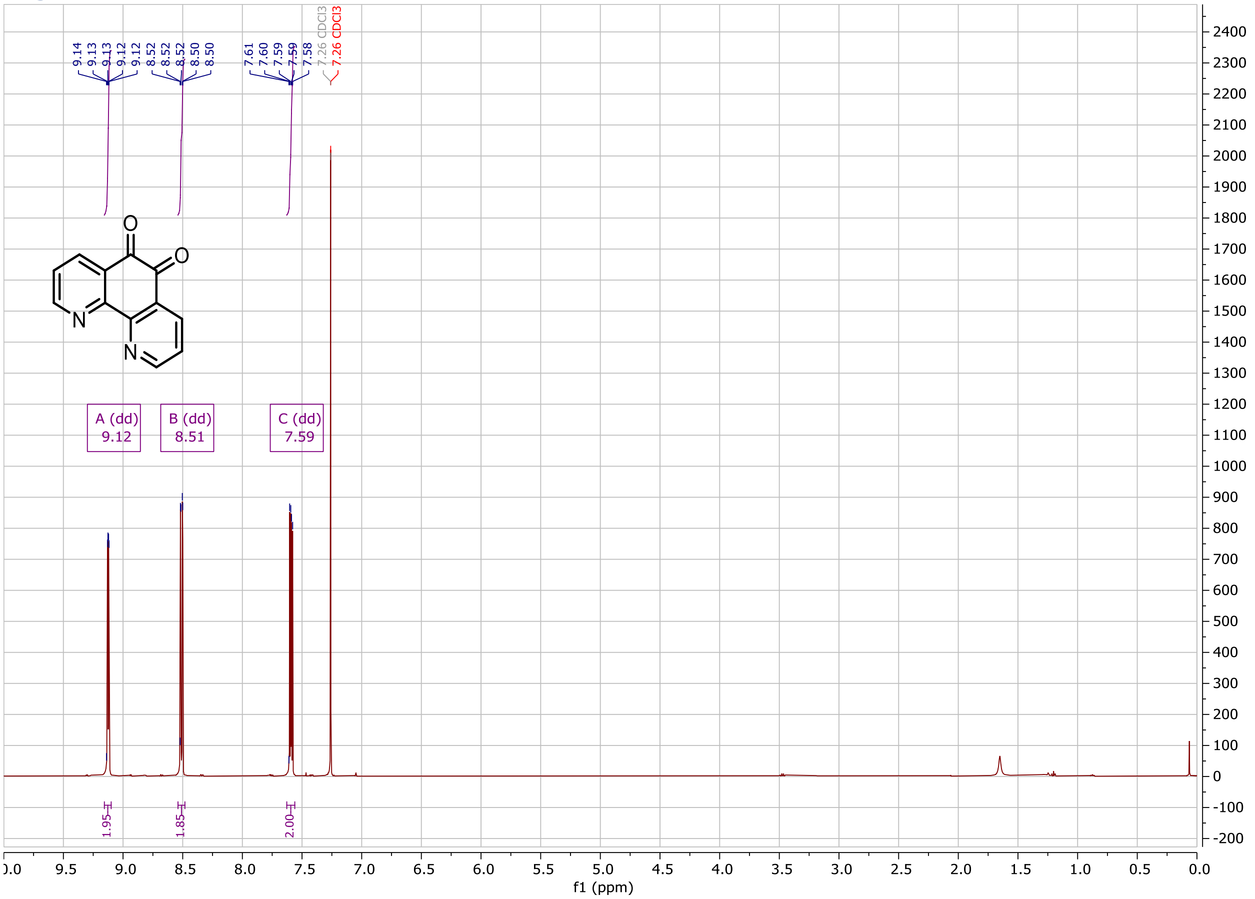

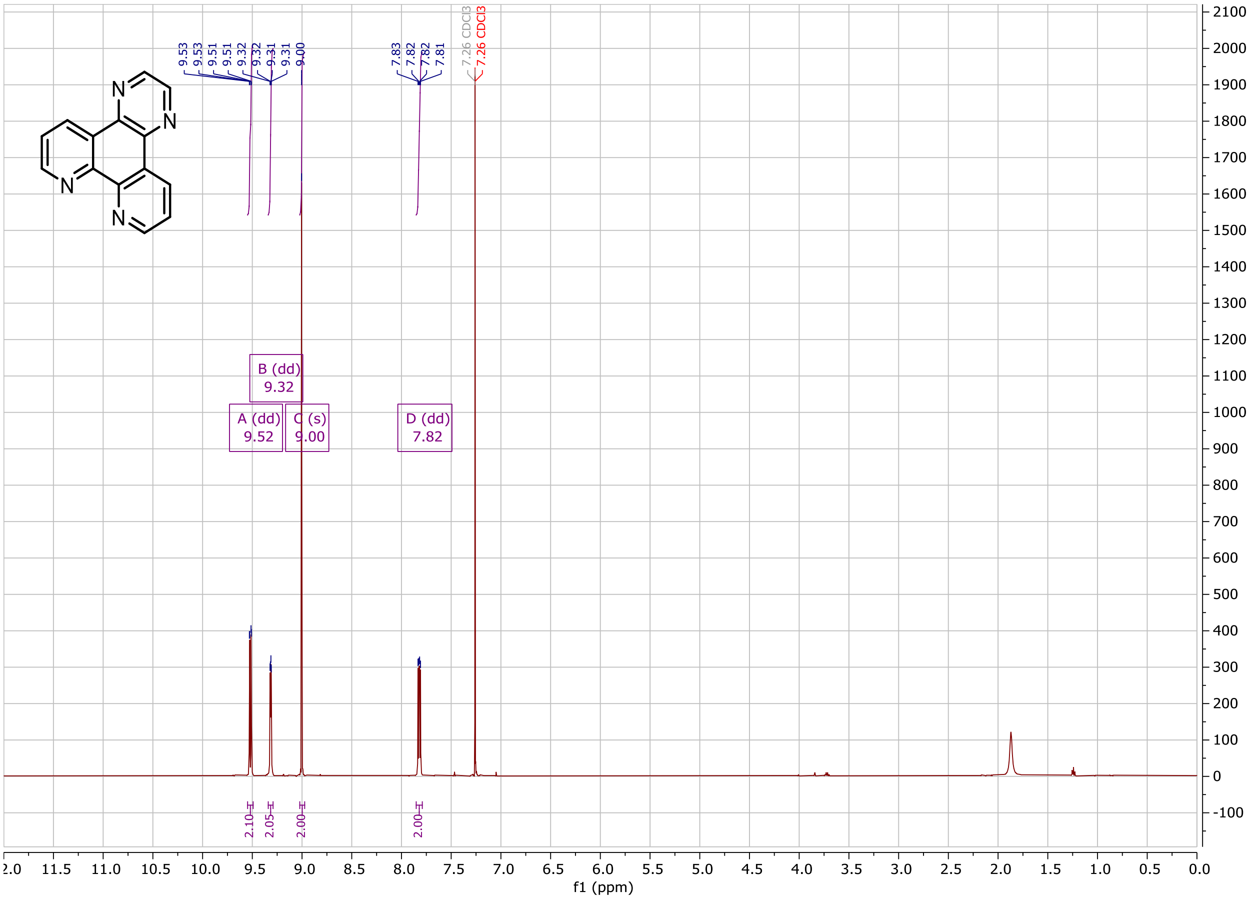

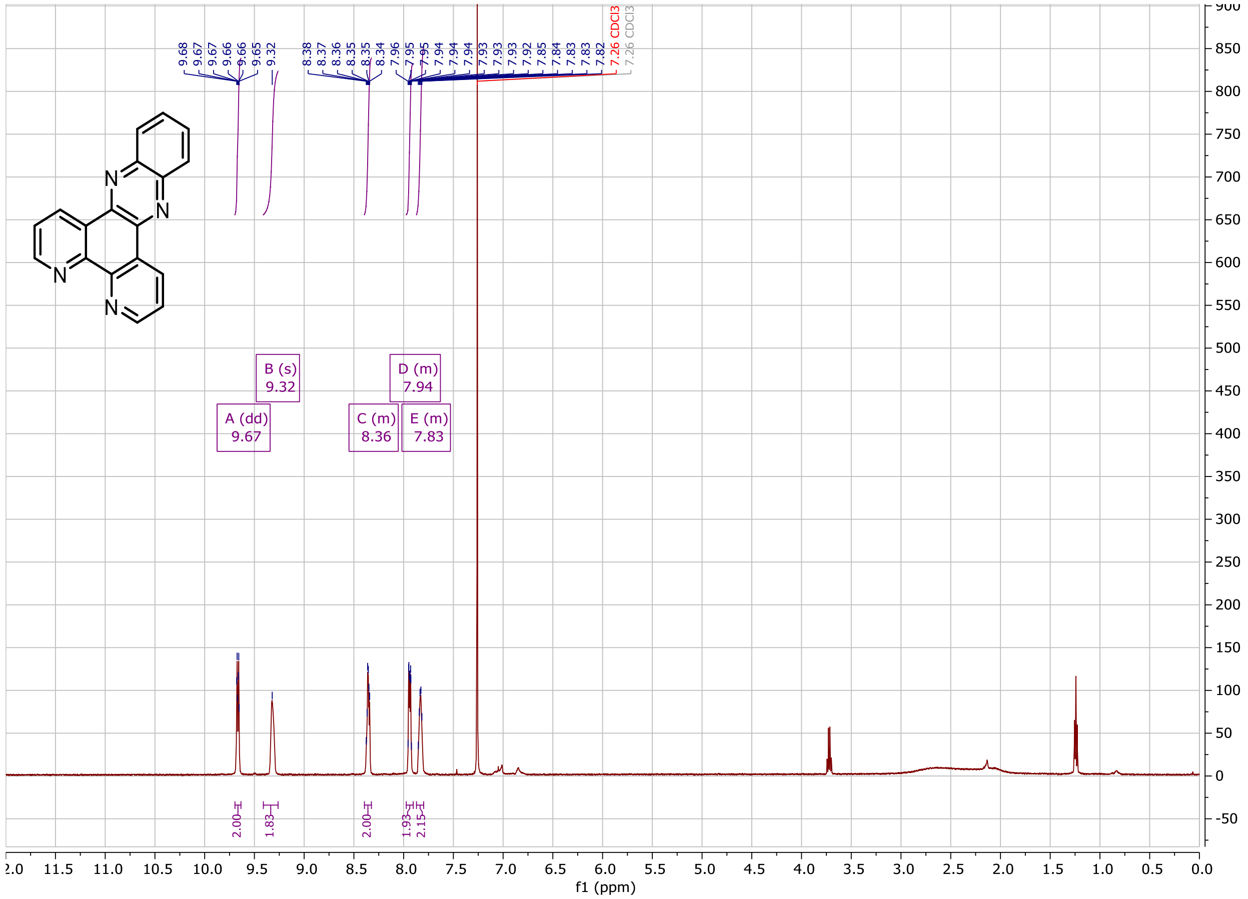
N
N
N
N
B (s)
9.32
A (dd)
9.67
D (m)
7.94
C (m)
8.36
E (m)
7.83
7.26 CDCl3
f1 (ppm)

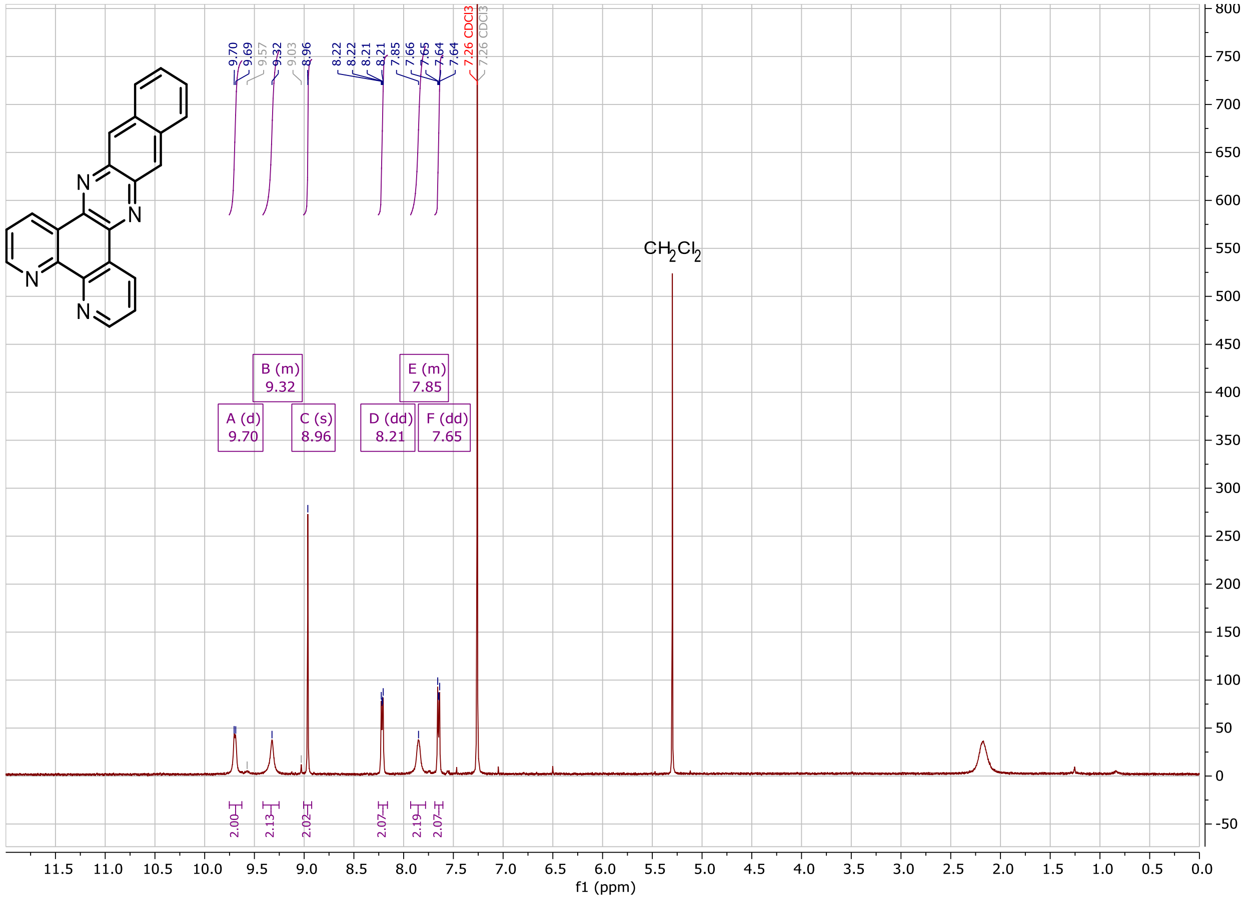
N
N
N
N
CH2Cl2
B (m)
9.32
A (d)
9.70
C (s)
8.96
E (m)
7.85
D (dd)
8.21
F (dd)
7.65
7.26 CDCl3
f1 (ppm)

Copper Complexes

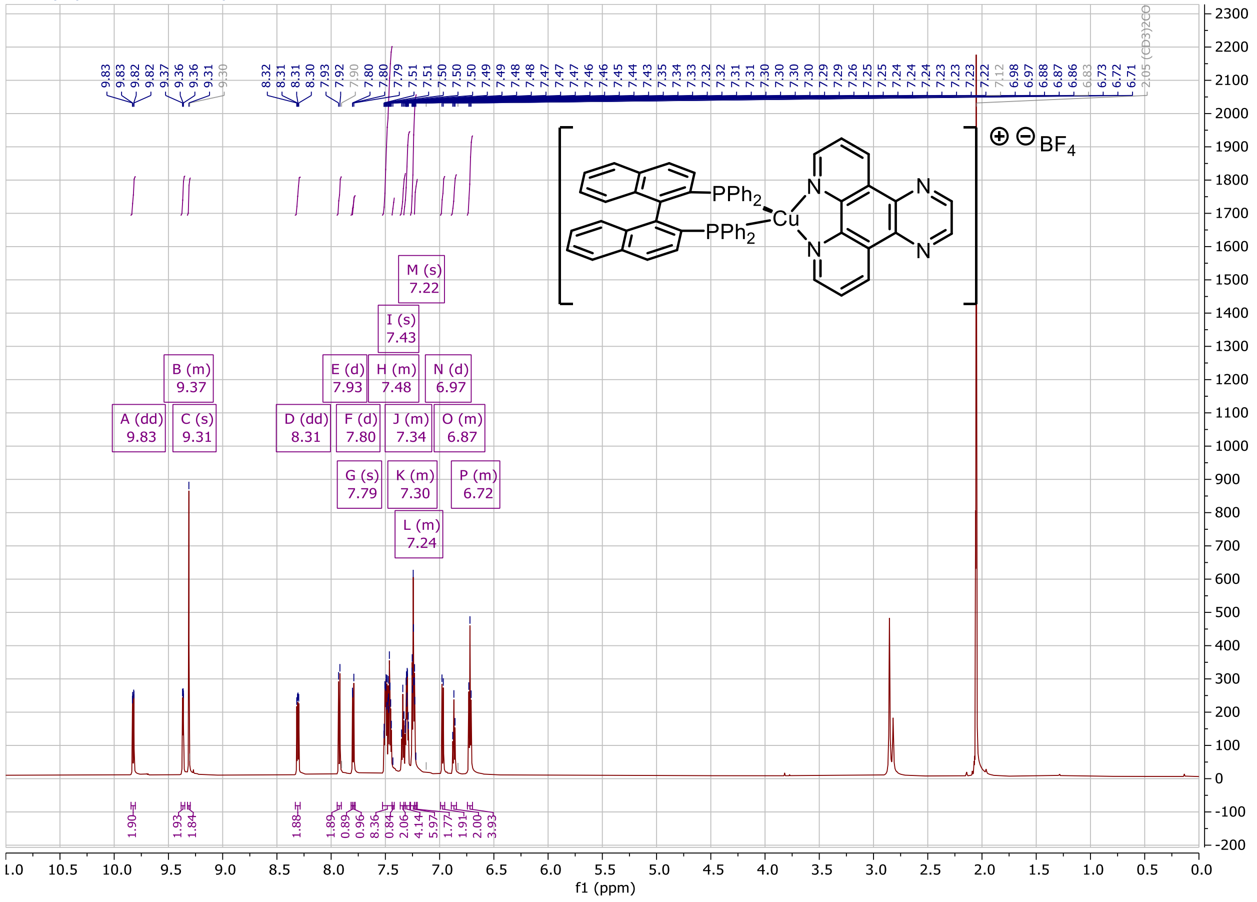

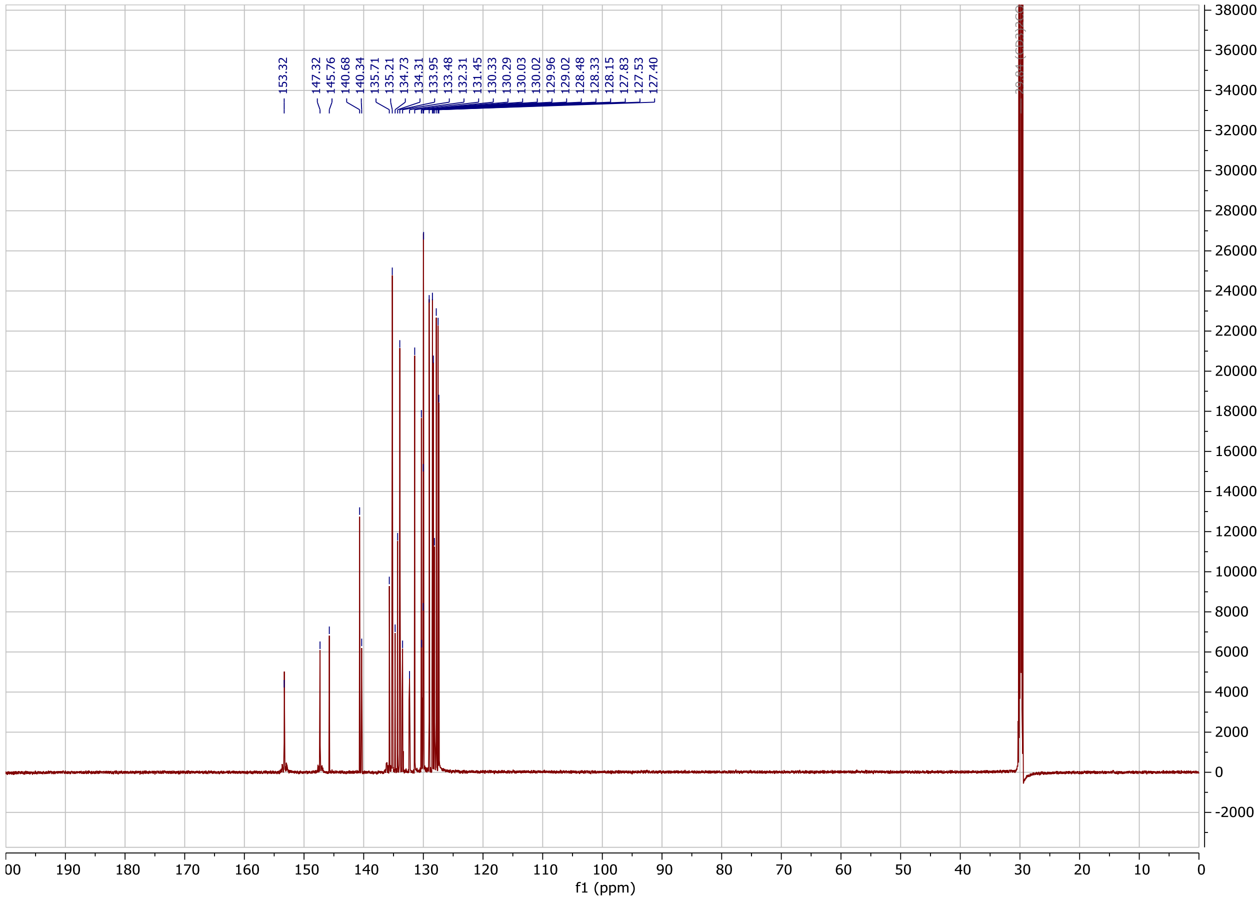

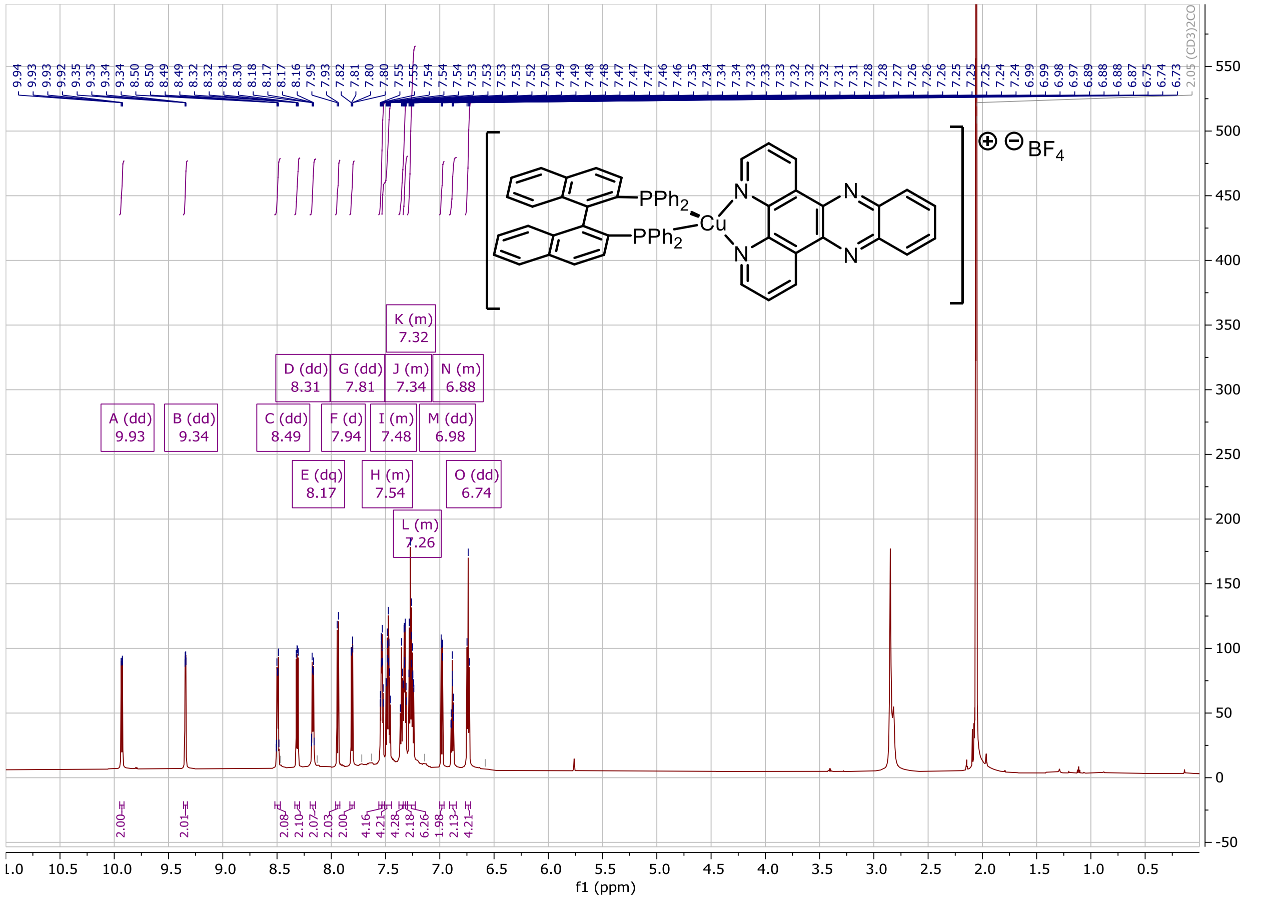

BF4
PPh2
PPh2
Cu
N
A (dd) 9.93
B (dd) 9.34
C (dd) 8.49
D (dd) 8.31
E (dq) 8.17
F (d) 7.94
G (dd) 7.81
H (m) 7.54
I (m) 7.48
J (m) 7.34
K (m) 7.32
L (m) 7.26
M (dd) 6.98
N (m) 6.88
O (dd) 6.74
2.00
2.01
2.08
2.10
2.07
2.03
2.00
4.16
4.21
4.28
2.18
6.26
1.98
2.13
4.21
f1 (ppm)

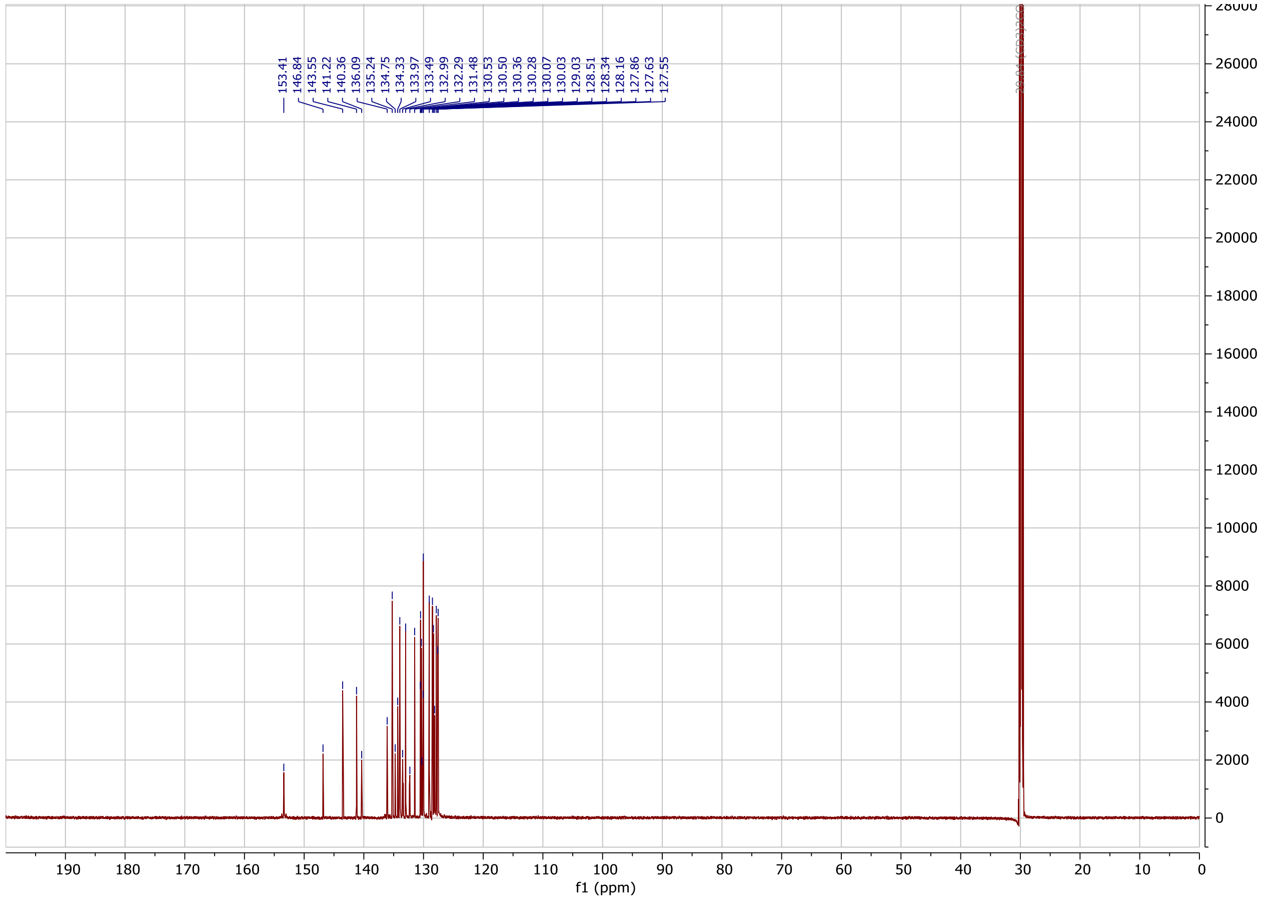

153.41
146.84
143.55
141.22
140.36
136.09
135.24
134.75
134.33
133.97
133.49
132.99
132.29
131.48
130.53
130.50
130.36
130.28
130.07
130.03
129.03
128.51
128.34
128.16
127.86
127.63
127.55
f1 (ppm)

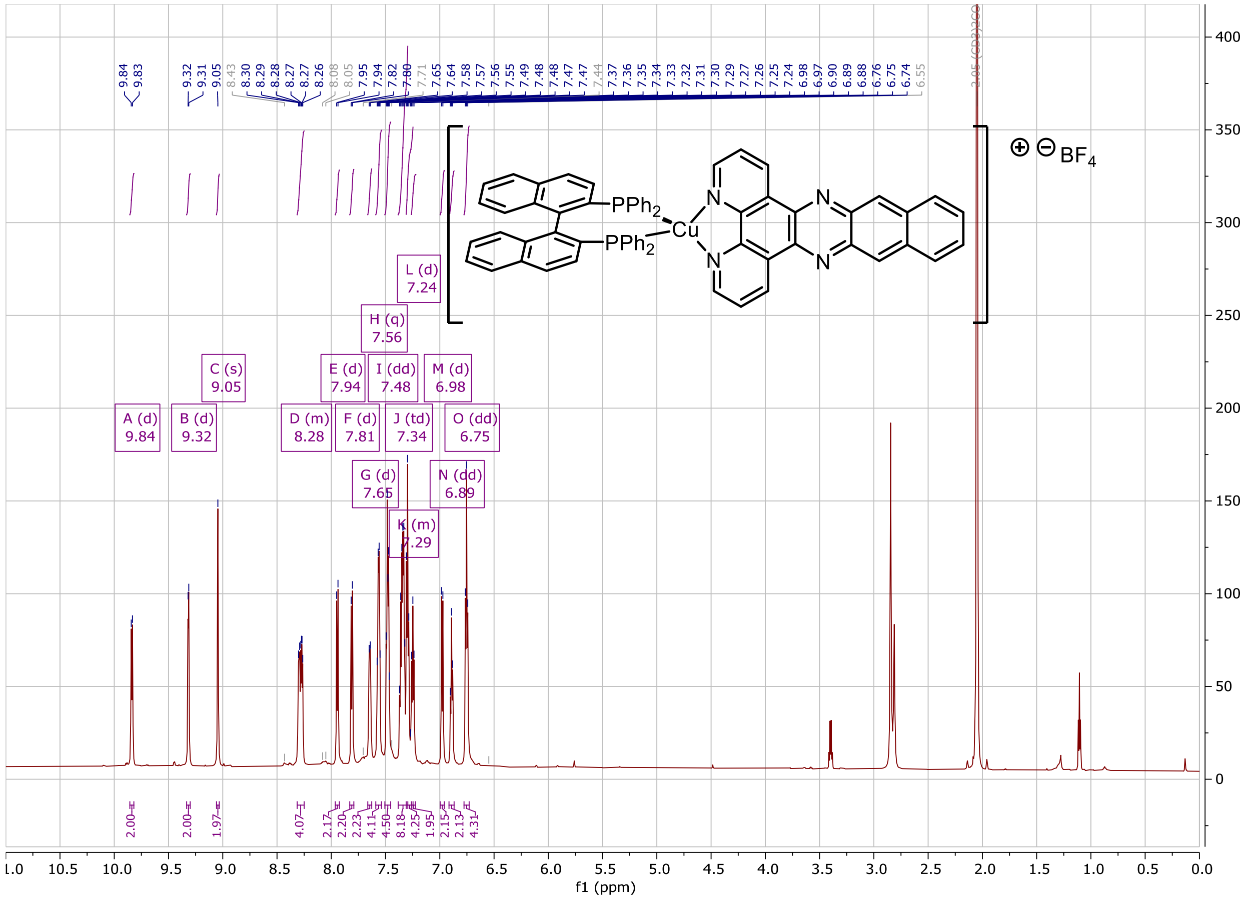
9.84
9.83
9.32
9.31
9.05
A (d)
9.84
B (d)
9.32
C (s)
9.05
D (m)
8.28
E (d)
7.94
F (d)
7.81
G (d)
7.65
H (q)
7.56
I (dd)
7.48
J (td)
7.34
K (m)
7.29
L (d)
7.24
M (d)
6.98
N (dd)
6.89
O (dd)
6.75
PPh2
Cu
N
BF4
f1 (ppm)

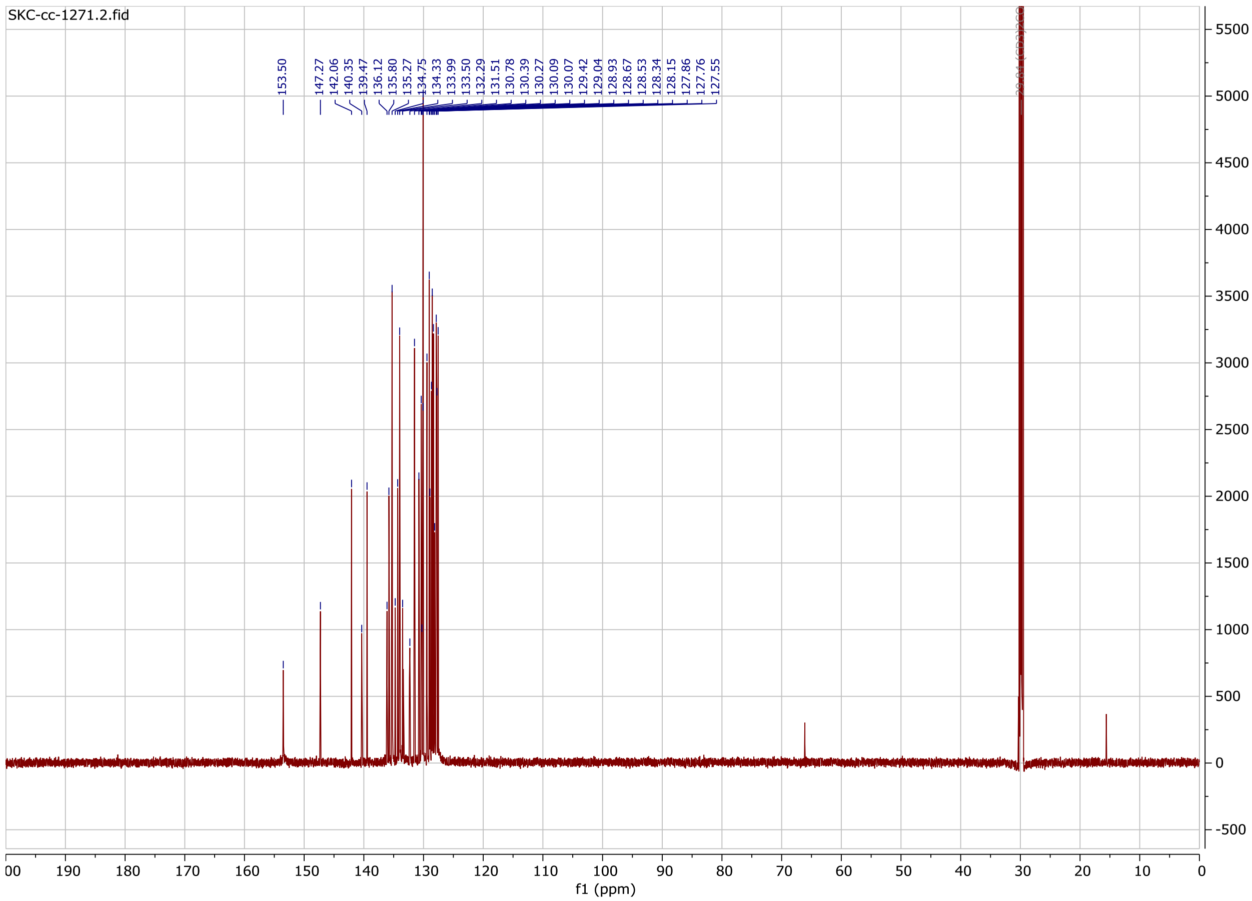
SKC-cc-1271.2.fid
153.50
147.27
142.06
140.35
139.47
136.12
135.80
135.27
134.75
134.33
133.99
133.50
132.29
131.51
130.78
130.39
130.27
130.09
130.07
129.42
129.04
128.93
128.67
128.53
128.34
128.15
127.86
127.76
127.55
f1 (ppm)

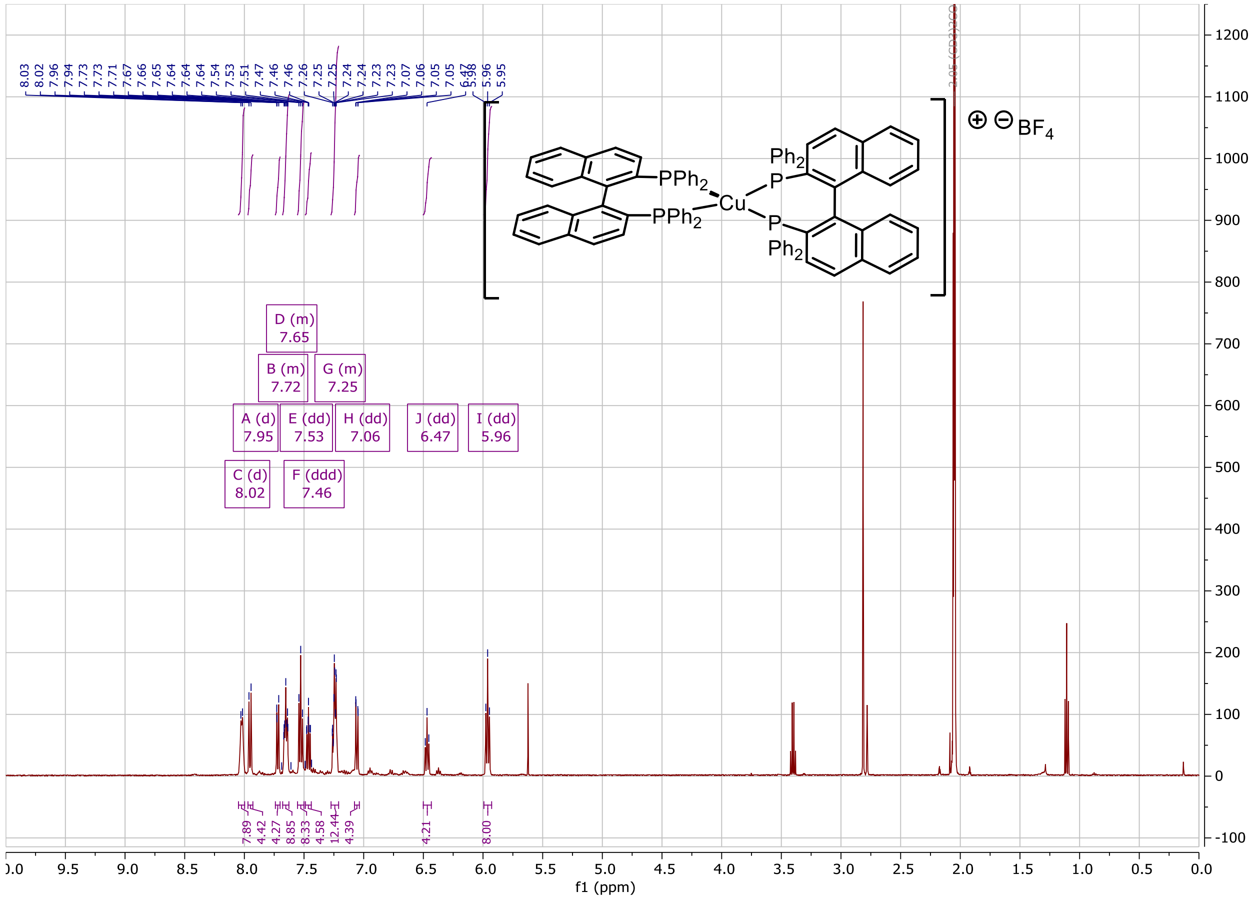
8.03 8.02 7.96 7.94 7.73 7.73 7.71 7.67 7.66 7.65 7.64 7.64 7.64 7.54 7.53 7.51 7.47 7.46 7.46 7.26 7.25 7.25 7.24 7.24 7.23 7.23 7.07 7.06 7.05 7.05 6.47 5.98 5.96 5.95
PPh2
Cu
Ph2
P
BF4
A (d) 7.95
B (m) 7.72
C (d) 8.02
D (m) 7.65
E (dd) 7.53
F (ddd) 7.46
G (m) 7.25
H (dd) 7.06
I (dd) 5.96
J (dd) 6.47
7.89 4.42 4.27 8.85 8.33 4.58 12.44 4.39 4.21 8.00
f1 (ppm)

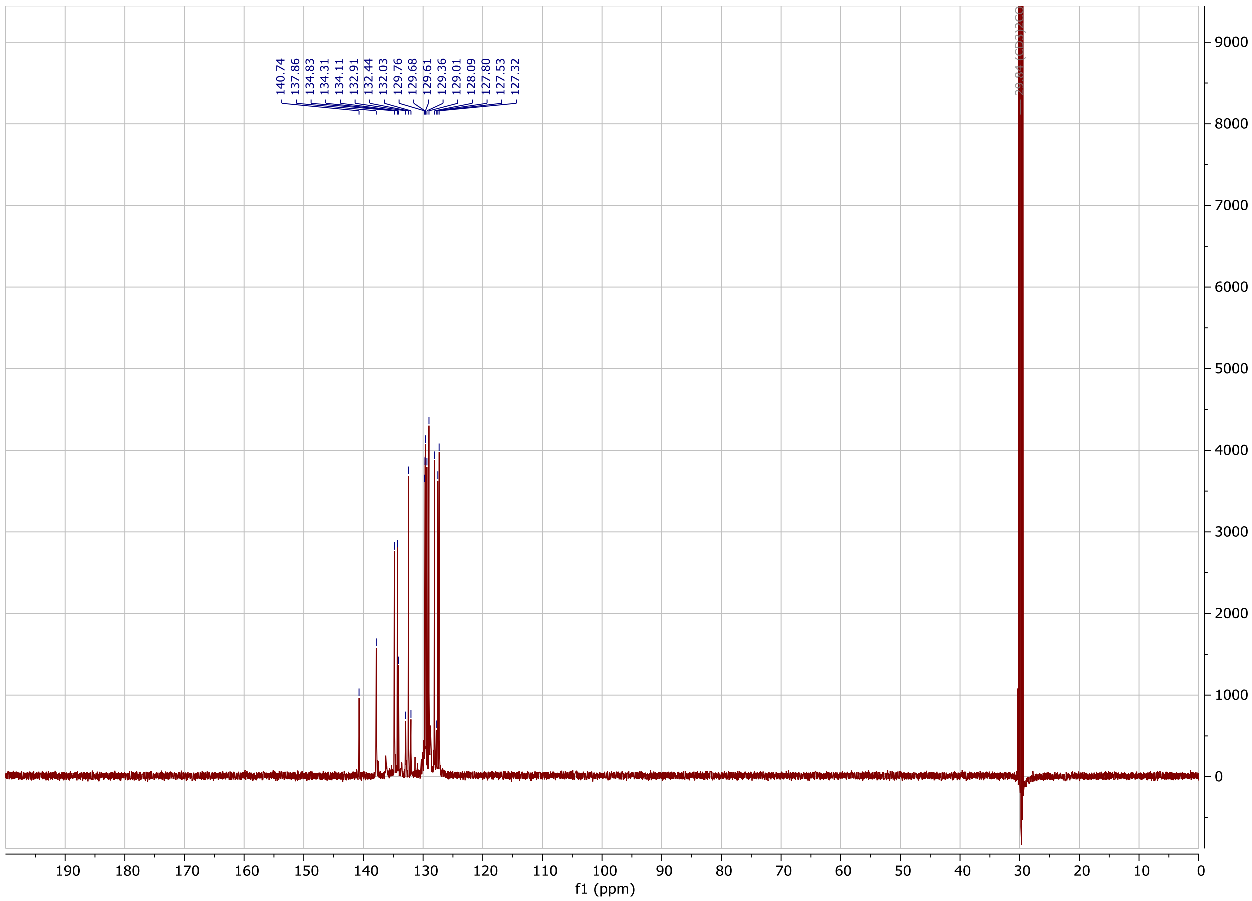
140.74 137.86 134.83 134.31 134.11 132.91 132.44 132.03 129.76 129.68 129.61 129.36 129.01 128.09 127.80 127.53 127.32
f1 (ppm)

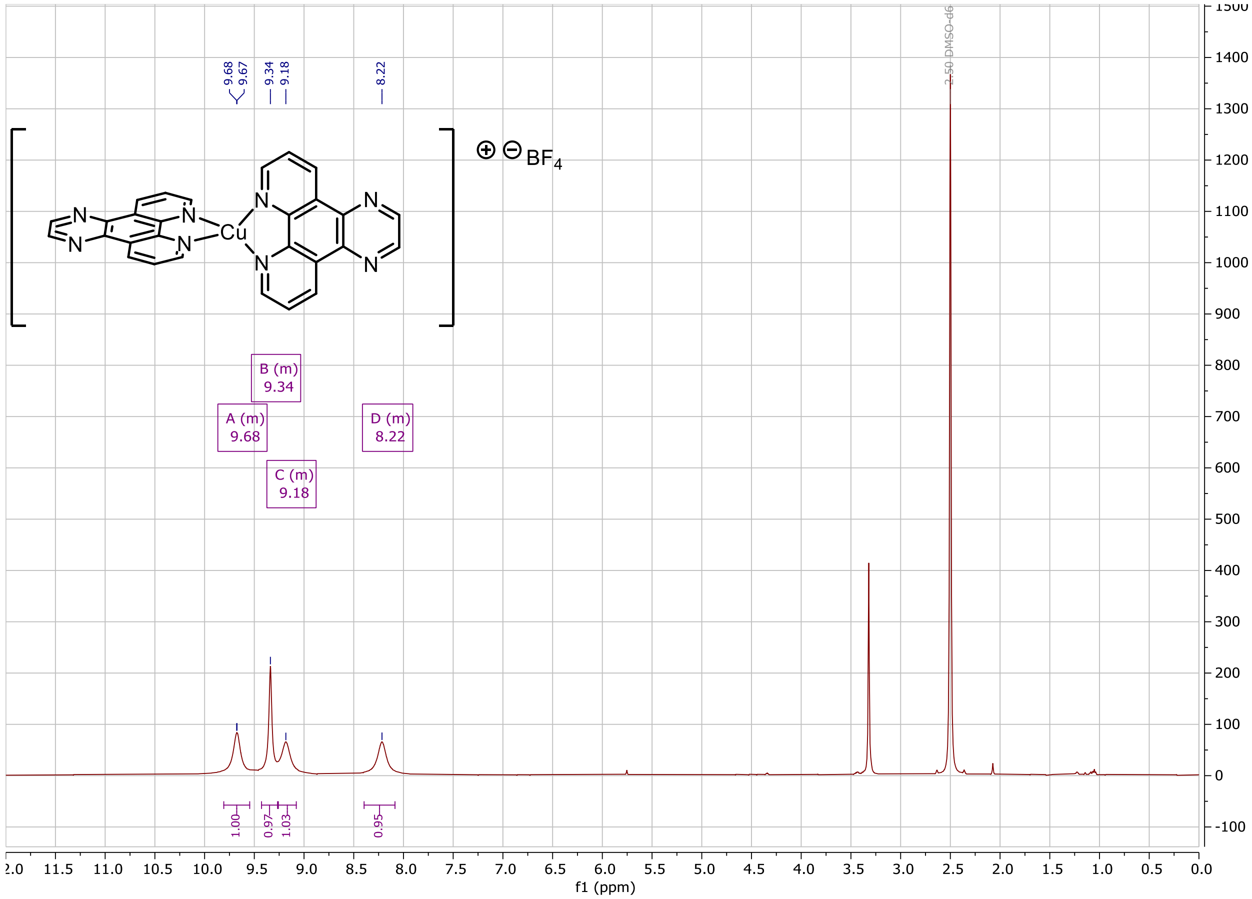
9.68
9.67
9.34
9.18
8.22
2.50 DMSO-d6
BF4
A (m) 9.68
B (m) 9.34
C (m) 9.18
D (m) 8.22
1.00
0.97
1.03
0.95
f1 (ppm)

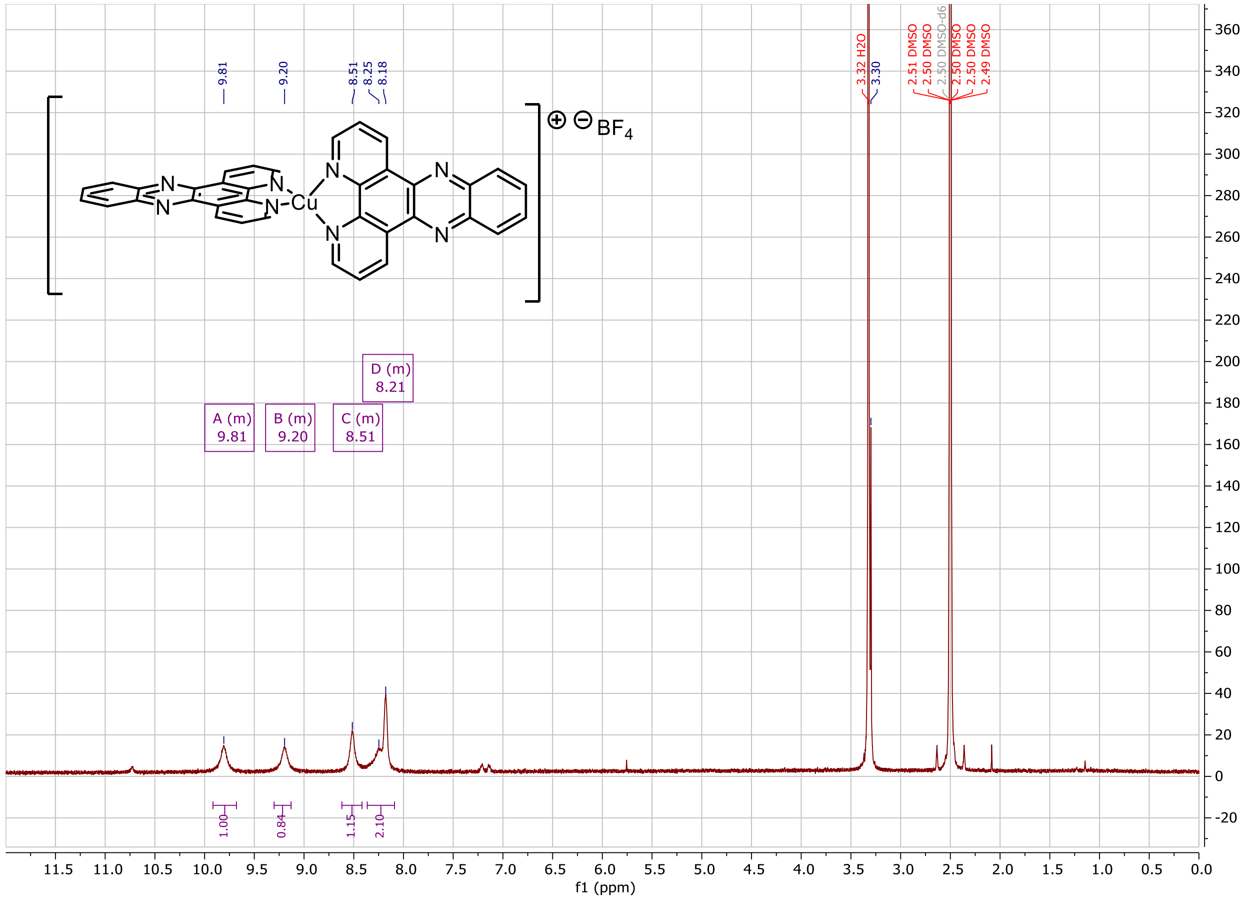
9.81
9.20
8.51
8.25
8.18
3.32 H2O
3.30
2.51 DMSO
2.50 DMSO
2.50 DMSO-d6
2.50 DMSO
2.50 DMSO
2.49 DMSO
BF4
A (m) 9.81
B (m) 9.20
C (m) 8.51
D (m) 8.21
1.00
0.84
1.15
2.10
f1 (ppm)
